# Spatial distribution and populations at risk of *A. lumbricoides* and *T. trichiura* co-infections and infection intensity classes: an ecological study

**DOI:** 10.1186/s13071-018-3107-y

**Published:** 2018-10-03

**Authors:** Kei Owada, Colleen L Lau, Lydia Leonardo, Archie C A Clements, Laith Yakob, Mark Nielsen, Hélène Carabin, Ricardo J Soares Magalhães

**Affiliations:** 1 School of Medicine, The University of Queensland, QLD, South Brisbane, Australia; 2 Children’s Health and Environment Program, Child Health Research Centre, The University of Queensland, QLD, South Brisbane, Australia; 3 Research School of Population Health, Australian National University, ACT, Canberra, Australia; 4 Department of Parasitology, College of Public Health, University of the Philippines Manila, Manila, Philippines; 5 Department of Disease Control, London School of Hygiene and Tropical Medicine, London, UK; 6 School of Psychology, The University of Queensland, QLD, St Lucia, Australia; 7 Faculty of Humanities, University of Johannesburg, Auckland Park, South Africa; 8 Department of Biostatistics and Epidemiology, College of Public Health, University of Oklahoma Health Sciences Center, Oklahoma, USA; 9 Spatial Epidemiology Laboratory, School of Veterinary Science, The University of Queensland, QLD, Gatton, Australia

**Keywords:** Soil-transmitted helminths, *Ascaris lumbricoides*, *Trichuris trichiura*, Co-infection, Infection intensity, Spatial epidemiology, Bayesian geostatistics, The Philippines

## Abstract

**Background:**

Soil-transmitted helminth (STH) infections are highly prevalent in the Philippines. Mapping the prevalence and high-intensity of STH co-infections can help guide targeted intervention programmes to reduce morbidity, especially among vulnerable school-aged children. In this study, we aimed to predict the spatial distribution of the prevalence of *Ascaris lumbricoides* and *Trichuris trichiura* co-infection and infection intensity classes in the Philippines to identify populations most in need of interventions.

**Methods:**

Data on STH infections from 29,919 individuals during the nationwide parasitological survey in 2005 to 2007 were included in the analysis. To geographically predict the prevalence of *A. lumbricoides* and *T. trichiura* co-infections and infection intensity classes, Bayesian multinomial geostatistical models were built including age, sex, environmental variables and a geostatistical random effect. The number of individuals co-infected and belonging to each of the infection intensity classes in 2017 was forecast by combining our predictive prevalence maps with population density maps.

**Results:**

Our models showed that school-aged children (5–19 years) are most at risk of *A. lumbricoides* and *T. trichiura* co-infections and of moderate/high infection intensity compared to other age groups. We identified target provinces where the likelihood of STH-associated morbidity was highest: Luzon (Bulacan, Benguet, Cavite, Sorsogon, Metropolitan Manila, Pampanga and Rizal), the Visayas (Cebu, Iloilo, Leyte and Negros Occidental), and in Mindanao (Agusan Del Norte, Davao Del Sur, Davao Oriental, Lanao Del Sur, Maguindanao, Misamis Oriental, Sulu and Zamboanga Del Sur). Luzon had the highest estimated number of school-aged children with *A. lumbricoides* and *T. trichiura* co-infections (estimated total 89,400), followed by the Visayas (38,300) and Mindanao (20,200).

**Conclusions:**

Our study provided epidemiological evidence to highlight national priority areas for controlling co-infections and high intensity infections in the Philippines. Our maps could assist more geographically targeted interventions to reduce the risk of STH-associated morbidity in the Philippines.

**Electronic supplementary material:**

The online version of this article (10.1186/s13071-018-3107-y) contains supplementary material, which is available to authorized users.

## Background

Soil-transmitted helminth (STH) infections with *Ascaris lumbricoides*, *Trichuris trichiura* and hookworm species including *Ancylostoma duodenale*, *Ancylostoma ceylanicum* and *Necator americanus* are among the most common infections in school-aged children. These infections are particularly common in impoverished communities where the provision of water, sanitation and hygiene education are limited [1]. Exposure to STH infections is also driven by environmental factors such as vegetation, land cover, elevation, temperature and water availability [2]. Additionally, sex and age have been shown to influence exposure and susceptibility to STH [3–5]. Morbidity caused by STH infections is estimated to incur 4.98 million years lived with disability (YLDs) for an estimated loss of 5.18 million disability-adjusted life years (DALYs) [1]. STH-related morbidity includes anaemia of inflammation and iron-deficiency, chronic nutritional imbalances, stunting, impaired cognitive function and delayed development of fine and gross motor functions [1, 6]. The highest burden for *A. lumbricoides* is observed in South and Southeast Asia and West Africa, and the highest relative burden for *T. trichiura* and hookworm is found in southern Africa [1]. Previous studies have demonstrated that STH-associated morbidity is exacerbated by co-infections and high intensity infections [2, 6–9].

To control STH morbidity, the World Health Organization (WHO) advocates the administration of regular chemotherapy with anthelminthic medicines such as albendazole and mebendazole to at-risk populations [6]. Importantly, the WHO proposes the global target of eliminating morbidity attributable to STH in school-aged children by 2020 by regularly treating at least 75% of school-aged children in STH endemic areas [10]. Treatment is given once a year where the prevalence of STH infections in the community is over 20%, and twice a year where the prevalence exceeds 50% [10] at the commencement of a programme.

The Philippine Department of Health has been implementing an integrated helminth control programme since 2006, using chemotherapy with albendazole or mebendazole, targeting all children aged 12 months to 12 years, pregnant women, adolescents, farmers and indigenous populations [11–13]. The integrated helminth control programme also includes health education approaches and installation of water and sanitation facilities [14]. However, even after one decade of integrated helminth control programme implementation, the WHO target of < 20% prevalence of STH infections and elimination of moderate and high intensity infections in school-aged children has not been reached in the Philippines [15–18]. According to the most recent WHO report, it is estimated that 19.6 million school-aged children live in STH endemic areas of the Philippines and require preventive chemotherapy [10].

The spatial distribution of STH infection prevalence shows significant variation across the Philippines [13, 19]. While mapping the prevalence of STH infections is important to define areas that need regular treatment with albendazole or mebendazole [17], the prevalence of infection alone is not necessarily a good indicator of population-level morbidity. Indicators based on co-infections and intensity of infection, which are linked to morbidity, might be more useful for estimating disease burden [4, 20]. Indeed, modelling the spatial distribution of STH infection intensity classes and co-infections has relevance for identifying communities where the likelihood of morbidity and parasite transmission is at its highest [4, 21]. Targeting treatment, prevention and control activities to communities with high-intensity STH infections, or co-infections, could lead to a more efficient reduction in transmission and severe STH-related morbidity compared to targeting treatment based only on prevalence of single infections [21–23]. Despite intervention schemes being implemented to reduce morbidity attributable to STH infections in the Philippines [11, 17], persistently high prevalence rates among school-aged children continue to be recorded across the country [15, 18, 24, 25]. Available evidence suggests that indicators based on co-infections and infection intensities, which are linked to higher degrees of morbidity, are useful for estimating disease burden. Furthermore, prediction maps based on co-infection and intensity models have made spatially targeted helminth control interventions possible [4, 26]. Studies of this kind have not been conducted in the Philippines. Therefore, in this study we attempt to apply this methodology to provide previously unavailable evidence such as predictive prevalence maps of STH co-infections and infection intensity classes to identify and quantify populations most at risk of STH associated morbidity in the Philippines and thus assist the local governments to focus their resource allocation on these areas.

## Methods

### Data for analysis

#### STH infection data

Information on study design, sample size and sampling procedures are detailed elsewhere [13]. In brief, we used STH infection data collected during the National Schistosomiasis Survey in the Philippines in 2005 to 2007 [3, 19]. Our previous study [1] used data collected from the National Schistosomiasis survey and from Western Samar [2]; however, the current study does not include the infection data from Samar due to the lack of reliability of the intensity of infection data. Therefore, to maintain data consistency across all models we only used the data collected by the National Schistosomiasis Survey. This brought the total number of individuals included in this study, after removing those without plausible intensity of infection information, to 2701 for Luzon, 7575 for the Visayas and 19,643 for Mindanao. In the national survey, STH infections were diagnosed by the detection of parasite eggs in two stool samples collected on two separate days from each individual, using a Kato-Katz thick smear examination; however, the submission of the second stool sample was reported to be inconsistent thus only the results from the first examination were taken [3, 19]. Infection intensity was expressed as eggs per gram of faeces (epg) [13]. Each individual was categorized into infected or not infected based on the presence of at least one egg. We only considered data for *A. lumbricoides* and *T. trichiura* because co-infections with these parasites were most prevalent (Additional file 1: Table S1).

We analysed infection intensity data only from Mindanao because the majority of STH infections for Luzon and the Visayas were of light infection intensity. We excluded hookworm from our infection intensity models because the majority of hookworm infections in Mindanao were of light infection intensity (1–1999 epg). Data on infection intensity were categorized according to the WHO classes, i.e. 0; 1–4999; 5000–49,999; and ≥ 50,000 epg for *A. lumbricoides*, and 0; 1–999; 1000–9999; and ≥ 10,000 epg for *T. trichiura* for light, moderate and high infection intensity, respectively [20]. We combined moderate and high infection intensity classes due to low prevalence of high infection intensity classes for all species of STH in our analyses (Table 1).

**Table 1:** Number of individuals with STH infections, and the distribution of intensity among those infected in Mindanao

STH species	Total number of individuals	Age group (in years)		
		Under 5	5–19	20 and older
*A. lumbricoides* ^a^				
With infection, *n*	2447	294	1249	904
Mean infection intensity (95% CI)	8450.8 (7574.1–9327.5)	15,163.9 (11,142.9–19,184.9)	9118.8 (7874.2–10,363.3)	5344.6 (4405.6–6283.7)
Light intensity, *n* (%)	1704 (69.6)	170 (57.8)	830 (66.5)	704 (77.9)
Moderate intensity, *n* (%)	651 (26.6)	99 (33.7)	365 (29.2)	187 (20.7)
High intensity, *n* (%)	92 (3.8)	25 (8.5)	54 (4.3)	12 (1.4)
*T. trichiura* ^b^				
With infection, *n*	2122	164	1064	894
Mean infection intensity (95% CI)	506.7 (384.9–628.5)	525.1 (332.5–717.6)	540.1 (380.2–699.9)	465.5 (250.2–680.8)
Light intensity, *n* (%)	1935 (91.2)	143 (87.2)	955 (89.7)	837 (93.6)
Moderate intensity, *n* (%)	176 (8.3)	21 (12.8)	104 (9.8)	51 (5.7)
High intensity, *n* (%)	11 (0.5)	0	5 (0.5)	6 (0.7)
Hookworm^c^				
With infection, *n*	1503	124	418	961
Mean infection intensity (95% CI)	275.0 (227.3–322.8)	368.4 (162.8–574.1)	243.5 (135.3–351.6)	276.7 (224.8–328.6)
Light intensity, *n* (%)	1473 (98.0)	121 (97.6)	412 (98.6)	940 (97.8)
Moderate intensity, *n* (%)	29 (1.3)	1 (0.8)	2 (0.5)	17 (1.8)
High intensity, *n* (%)	10 (0.7)	2 (1.6)	4 (0.9)	4 (0.4)

^a^Light infection intensity: 1–4999 epg; moderate: 5000–49,999 epg; high: ≥ 50,000 epg [20]

^b^Light infection intensity: 1–999 epg; moderate: 1000–9999 epg; high: ≥ 10,000 epg [20]

^c^Light infection intensity: 1–1999 epg; moderate: 2000–3999 epg; high: ≥ 4000 epg [20]

Data from the regions of Luzon and the Visayas were combined due to the size of the separate datasets, the frequent movement of people between islands of both regions and the similar spatial dependence patterns in these two regions as demonstrated in our spatial analyses. The presence of infection was measured for a total of 10,276 participants for Luzon and the Visayas, and 19,643 for Mindanao, from 187 survey locations (i.e. 79 locations in Luzon and the Visayas; 108 locations in Mindanao), with complete information regarding STH infection status, barangay geolocation and demography (i.e. age and sex). For the purpose of this study, age was stratified as categorical variables (children under 5 years-old, school-aged children defined as 5–19 years-old, and individuals aged 20 years and older).

#### Environmental data

Environment data for land surface temperature (LST), rainfall and distance to perennial water bodies (DPWB) were obtained from WorldClim (https://www.worldclim.org). Normalized difference vegetation index (NDVI) data, which serves as a proxy measure of rainfall for a 1 × 1 km grid cell resolution, were obtained from the National Oceanographic and Atmospheric Administrations’ (NOAA) Advanced Very High Radiometer [27]. Values of these environmental covariates were extracted in ArcGIS version 10.4.0.5524 [28] for each barangay.

### Spatial analysis

#### Geo-referencing of data

The unit of analysis was the barangay (the smallest administrative unit in the Philippines), which has an average diameter of approximately 11 km. Barangay centroids were estimated using the geographical information system (GIS) software Quantum GIS version 1.7.3 (QGIS Development Team, 2011) based on a shapefile of the barangays of the Philippines, obtained from the geographical data warehouse DIVA GIS (https://www.diva-gis.org/Data) and PhilGIS (https://www.philgis.org). Infection data and environmental data were linked to the barangay centroid.

#### Variable selection

In all models, initial variable selection included sex and age, and environmental variables (rainfall, DPWB, LST and NDVI). LST were considered in the analysis because *A. lumbricoides* and *T. trichiura* have thermal thresholds outside of which the survival of the infective stages in the soil declines [4, 29]. Rainfall, NDVI and DPWB were also considered because these affect the moisture of the soil where the helminth infective stages are found, and therefore their survival. Correlations between environmental covariates were investigated using Pearson’s correlation coefficients.

Frequentist fixed-effects multinomial regression models of *A. lumbricoides* and *T. trichiura* co-infections were developed. We used multinomial models for the prevalence of STH infection intensity classes rather than infection intensity data on a continuous scale. This was done to facilitate estimation of the number of population with a particular infection intensity class. The interaction between age and sex was checked in all models using the “*mfpigen*” command in Stata version 13.1 [30]. It was found that an interaction term between age and sex improved the STH co-infection model for Mindanao only. Previous studies indicated that the non-linear relationship between infection and environmental covariates could be addressed parametrically [4, 26]. In all models, non-linearity between STH infection outcomes and environmental data was investigated [4]. Quadratic terms for DPWB and rain were found to improve model fit of non-spatial multinomial models for the prevalence of *A. lumbricoides* infection intensity classes, as per the Akaike’s Information Criterion (AIC) [31]. Covariates were included in the final model based on backwards-stepwise regression analysis (with Wald’s *P* > 0.2 as the exclusion criterion, and *P* < 0.05 as the entry criterion).

#### Analysis of residual spatial dependence

Residuals for non-spatial models with the final set of covariates were extracted and examined for spatial autocorrelation by generating semivariograms using the *geoR* package of R software version 3.1.1 (The R foundation for statistical computing, Vienna, Austria, https://www.r-project.org). Further detailed information regarding analysis and results of residual spatial dependence are provided in detail in Additional file 1: Text S1. The semivariograms for Luzon and the Visayas (Additional file 1: Figure S1) demonstrated similar spatial dependence, justifying the combining of data from these two regions into a single region for subsequent modelling.

#### Spatial models

Multinomial models with geostatistical random effects were built using OpenBUGS (MRC Biostatistics Unit, Cambridge, and Imperial College London, UK) for Luzon/the Visayas and Mindanao.

In our co-infection models, survey data were aggregated into groups according to age, sex, and location using four infection outcomes (i.e. no infection; *A. lumbricoides* monoinfection; *T. trichiura* monoinfection; and *A. lumbricoides* and *T. trichiura* co-infection). For the Mindanao STH infection intensity class models, we used three infection intensity outcomes for *A. lumbricoides* and *T. trichiura* infections (i.e. no infection; light intensity; and moderate/high intensity). All models included an intercept, individual-level variables such as age (categorized into three age groups: aged < 5 years; 5–19 years; and ≥ 20 years) and sex, the selected environmental variables, and a geostatistical random effect. Due to computational demands, our spatial models for Mindanao did not include quadratic terms for rainfall and DPWB variables (in the infection intensity model for *A. lumbricoides*) or interaction terms between age and sex (in the *A. lumbricoides* and *T. trichiura* infection intensity models and co-infection model). For all models, the reference category was “No infection”.

#### Formal model presentation

For co-infection models and infection intensity class models, we assumed:

$$ {Y}_{ijk}\sim Multinomial\left({p}_{ijk,}{n}_{ijk}\right), and\kern0.5em {p}_{ijk}=\frac{\phi_{ijk}}{\sum \limits_k{\phi}_{ijk}} $$

where *Y*_*ijk*_ is the observed number of children at location *i* in age-sex group *j* and outcome group *k*. For co-infection models, *i =* 1,..,187, *j =* 1,…,6, and *k =* 1,…,4. The 187 locations come from 79 locations in Luzon and the Visayas, and 108 locations in Mindanao. The six sex-age groups were made of male/female and ages < 5 years, 5–19 years, and ≥ 20 years. The four outcome groups were: No infection (reference group); *A. lumbricoides* monoinfection; *T. trichiura* monoinfection; and *A. lumbricoides* and *T. trichiura* co-infection. In contrast to co-infection models, for infection intensity class models, *i =* 1,..,108, *j =* 1,…,6, and *k =* 1,…,3. The 108 locations come from the survey locations in Mindanao alone. The six sex-age groups were the same as for co-infection models. The three outcome groups were: No infection (reference group); light infection intensity; and moderate/high infection intensity. *n*_*ijk*_ is the number tested and *p*_*ijk*_ is the probability of infection. Here *Φ*_*ijk*_ can be thought of as the overall odds of being in a specific outcome group relative to not being infected. To give a reference value, *Φ*_*ij1*_ (for the no infection group) was constrained to equal 1. The nominal regression models were fitted for the other outcome groups for both co-infection models and infection intensity class models as:

$$ \log \left({\phi}_{ijk}\right)=\kern0.5em {\alpha}_k+\kern0.5em \sum \limits_{z=1}^T{\beta}_{zk}\kern0.5em \times \kern0.5em {X}_{zij}\kern0.5em +\kern0.5em {\theta}_{ik} $$

where *α*_*k*_ is the outcome group-specific intercept, *β* is a matrix of *T* coefficients and *X* is a matrix of *T* covariates. The *θ*_*ik*_ are coefficients representing location-level (i.e. barangay) spatial effects for the prevalence of each mono- and co-infection and of each infection intensity class. They have a multivariate normal distribution *θ*_*ik*_~*MVN*(0, ∑_*ik*_) and are defined by isotropic powered exponential spatial correlation functions:

$$ {\sum}_{ik}=\kern0.5em f\left({d}_{ab},\phi \right)\kern0.5em =\kern0.5em {\sigma}^2\exp \left[-\left(\phi {d}_{ab}\right)\right] $$

where *d*_*ab*_ are the distances between pairs of survey locations *a* and *b*, the parameter phi (*Φ*) is the rate of decay of spatial correlation per unit of distance, and indicates the size of clusters, and σ^2^ is a variance parameter. For the Mindanao model, non-informative priors were used for the intercept alpha (*α*), and the effect sizes of covariates beta *(β)* (normal distribution prior with mean zero and precision 0.001). For the Luzon and Visayas model, the prior for the intercept and the effect size of covariates had mean zero and precision 0.01. The geostatistical random effects were assumed to follow a normal distribution, with a mean of zero and a variance of 1/*ui*, where the precision of *ui* was given a non-informative gamma prior distribution with shape and scale parameters = 2, 0.05. The prior distribution of *Φ* was also given a non-informative gamma prior distribution with shape and scale parameters = 2, 0.05 [26]. The radius of a cluster measured in decimal degrees corresponds to 3/*Φ*. One decimal degree is equivalent to approximately 111 km at the equator (the radii of cluster = 3/*Φ* × 111 km - the further away from the equator the multiplier increases).

#### Prediction maps

Model predictions were used to generate representative risk maps of the prevalence of *A. lumbricoides* and *T. trichiura* monoinfections and co-infections across the Philippines, and the prevalence of infection intensity classes across Mindanao region, for females aged 5–19 years, the subgroup with the highest prevalence of monoinfection, co-infections and infection intensity classes (note: all other age and sex groups would have a different overall mean infection prevalence, but the same spatial patterns of infection risk). Predictions were made at the nodes of a 0.05 × 0.05 decimal degree grid (approximately 5 km^2^). The mean and standard deviation were extracted from the posterior distributions of predicted risk and plotted in ArcGIS version 10.4.0.5524 [28]. Further detailed information regarding model specification is provided in Additional file 1: Text S2.

#### Model validation

The WHO recommends that in order to mitigate STH morbidity, an area must attain prevalence less than 20% as well as eliminate moderate/high infection intensities entirely [32]. The models were validated in terms of their ability to predict prevalence of higher or lower than 20% as per WHO guidelines.

The area under the curve (AUC) of the receiver operating characteristic (ROC) was used to determine discriminatory performance of the model predictions relative to observed co-infections prevalence thresholds of 20% at validation locations. Validation was indeed based on holding a random 25% subset of the data and performing AUC ROC to assess the discriminatory ability of the model on the 20% WHO threshold of prevalence. AUC ROC is a valid model validation procedure routinely used in similar studies [13]. For our models of prevalence of STH infection intensity classes, we used the mean prevalence of infection intensity classes of *A. lumbricoides* and *T. trichiura* as our cut-off value to determine discriminatory performance of the model predictions (20% and 15%, respectively). An AUC of 0.50–0.69 was taken to indicate poor discriminative ability; 0.70–0.89 reasonable discriminative ability; and ≥ 0.90 good discriminatory ability [33].

### Estimation of the number of infections in population most at risk of STH-associated morbidity in the Philippines in 2017

School-aged children (5–19 years old) were identified as being most at risk of STH-associated morbidity in our analysis. In order to estimate the number of school-aged children aged 5–19 years at risk, we multiplied a raster map of the estimated total number of school-aged children in 2017 by the predicted prevalence of *A. lumbricoides* and *T. trichiura* monoinfection and co-infections, and the prevalence of infection intensity classes. To derive the 2017 population raster map, we multiplied the 2015 population raster map from the AsiaPop project [34] by the reported UNDP annual population growth rate for 2015–2020, which was retrieved from the World Population Prospects 2015 Revision Population Database [35]. To generate a raster map of the estimated total number of school-aged children in 2017 in the Philippines, the 2017 population raster map was multiplied by the proportion of the Filipino population aged 5–19 years to derive a map of the number of school-aged children/km^2^, and then summed by region. All estimates were conducted in the ArcGIS Map algebra raster calculator [28].

## Results

### Descriptive findings

For the purpose of spatial modelling, 10,276 individuals in the combined region (i.e. Luzon and the Visayas) and 19,643 individuals in Mindanao with complete information were included in the analysis (Additional file 1: Table S2). The observed prevalence of infection was higher in females than males in all regions. Among children aged under 5 years and school-aged children (5–19 years), the prevalence of *A. lumbricoides* and *T. trichiura* co-infections was highest in Luzon and the Visayas, whereas the prevalence of *A. lumbricoides* monoinfection was highest in Mindanao. *Trichuris trichiura* monoinfection was highest in individuals aged 20 and older in Luzon and the Visayas. *Ascaris lumbricoides* monoinfection was highest among this age group in Mindanao. In the case of the infection intensity (Mindanao only), among those infected, the majority of *A. lumbricoides* and *T. trichiura* infection intensity classes were of light infection intensity; 30% were of moderate/high infection intensity classes of *A. lumbricoides* and, approximately 9% were of moderate/high intensity of *T. trichiura* infections.

### Spatial risk prediction

In Luzon, the Visayas, and Mindanao, females had significantly higher risk of *A. lumbricoides* monoinfection and *A. lumbricoides* and *T. trichiura* co-infections compared to males (Tables 2, 3). Similar results were found in each infection intensity class for both *A. lumbricoides* and *T. trichiura* infections (Table 4).

**Table 2:** Spatial effects for the prevalence of mono- and co-infections in Luzon and the Visayas

Coefficient	*A. lumbricoides* mono mean (95% BCI)^a^	*T. trichiura* mono mean (95% BCI)^a^	Co-infection mean (95% BCI)^a^
Female *vs* male	0.38 (0.23–0.52)	0.07 (-0.05–0.19)	0.18 (0.05–0.30)
Age 5–19 *vs* < 5 years-old	0.54 (0.34–0.75)	1.21 (1.01–1.42)	1.18 (0.98–1.37)
Age ≥ 20 *vs* < 5 years-old	-0.10 (-0.30–0.11)	0.79 (0.58–0.99)	0.07 (-0.13–0.27)
Normalised difference vegetation index^b^	0.10 (-0.15–0.36)	0.046 (-0.30–0.43)	0.24 (-0.26–0.76)
Temperature^b^	-0.16 (-0.39–0.08)	-0.19 (-0.51–0.15)	-0.16 (-0.53–0.22)
Intercept	-1.93 (-2.23– -1.65)	-1.98 (-2.46– -1.55)	-1.97 (-2.54– -1.48)
Phi^c^	47.96 (4.66–97.51)	10.69 (2.53–59.88)	10.04 (2.40–60.54)
Tau, precision	1.11 (0.70–1.65)	0.60 (0.36–0.90)	0.35 (0.21–0.51)

^a^*BCI* Bayesian Credible Interval (The posterior distributions are summarized by the posterior mean and 95% BCI. A variable was considered as influencing the outcome if it excluded 0)

^b^Variables were standardized to have mean of zero, and standard deviation of 1

^c^Rate of decay of spatial autocorrelation, measured in decimal degrees; 3/phi determines the cluster size; one decimal degree is approximately 111 km at the Equator (the size of the radii of the clusters)

**Table 3:** Spatial effects for the prevalence of mono- and co-infections in Mindanao

Coefficient	*A. lumbricoides* mono mean (95% BCI)^a^	*T. trichiura* mono mean (95% BCI)^a^	Co-infection mean (95% BCI)^a^
Female *vs* male	0.11 (0.02–0.19)	0.12 (0.01–0.23)	0.19 (0.07–0.32)
Age 5–19 *vs* < 5 years-old	0.24 (0.11–0.38)	1.39 (1.16–1.63)	1.39 (1.20–1.59)
Age ≥ 20 *vs* < 5 years-old	-0.43 (-0.57– -0.29)	0.92 (0.70–1.16)	0.30 (0.10–0.51)
Normalised difference vegetation index^b^	-0.01 (-0.21–0.19)	-0.23 (-0.53–0.06)	-0.52 (-0.86– -0.15)
Intercept	-1.87 (-2.08– -1.65)	-4.88 (-5.27– -4.38)	-3.57 (-3.97– -3.19)
Phi^c^	65.9 (23.67–98.44)	0.10 (0.10–0.12)	50.6 (13.35–96.77)
Tau, precision	1.23 (0.88–1.65)	0.01 (0.01–0.02)	0.39 (0.27–0.53)

^a^*BCI* Bayesian Credible Interval (The posterior distributions are summarized by the posterior mean and 95% BCI. A variable was considered as influencing the outcome if it excluded 0)

^b^Variables were standardized to have mean of zero, and standard deviation of 1

^c^Rate of decay of spatial autocorrelation, measured in decimal degrees; 3/phi determines the cluster size; one decimal degree is approximately 111 km at the Equator (the size of the radii of the clusters)

**Table 4:** Spatial effects for the prevalence of infection intensity classes in Mindanao

Coefficient	*A. lumbricoides* light intensity (95% BCI)^a^	*A. lumbricoides* moderate/high intensity (95% BCI)^a^	*T. trichiura* light intensity (95% BCI)^a^	*T. trichiura* moderate/high intensity (95% BCI)^a^
Female *vs* male	0.16 (0.05–0.27)	0.38 (0.20–0.57)	0.12 (0.02–0.22)	0.49 (0.15–0.86)
Age 5–19 *vs* < 5 years-old	0.72 (0.51–0.91)	1.11 (0.85–1.37)	1.26 (1.05–1.47)	3.17 (2.43–3.93)
Age ≥ 20 *vs* < 5 years-old	0.18 (-0.02–0.37)	0.06 (-0.22–0.35)	0.77 (0.57–0.99)	2.16 (1.34–2.98)
Distance to perennial water bodies^b^	-0.71 (-1.19– -0.03)	0.43 (-0.37–1.4)	ni	ni
Rain^b^	0.42 (-0.45–1.05)	0.15 (-0.69–1.05)	ni	ni
Normalised difference vegetation index^b^	ni	ni	0.18 (-0.18–0.57)	-0.36 (-0.87–0.16)
Intercept	-5.38 (-6.12– -4.33)	-6.36 (-6.92– -5.71)	-4.19 (-4.53– -3.76)	-6.98 (-7.75– -6.02)
Phi^c^	4.80 (1.89–10.55)	4.52 (1.56–9.63)	4.52 (1.69–8.88)	20.38 (1.14–86.09)
Tau, precision	0.10 (0.05–0.17)	0.13 (0.05–0.23)	0.23 (0.11–0.38)	0.72 (0.21–1.61)

^a^*BCI* Bayesian Credible Interval (The posterior distributions are summarized by the posterior mean and 95% BCI. A variable was considered as influencing the outcome if it excluded 0)

^b^Variables were standardized to have mean of zero, and standard deviation of 1

^c^Rate of decay of spatial autocorrelation, measured in decimal degrees; 3/phi determines the cluster size; one decimal degree is approximately 111 km at the Equator (the size of the radii of the clusters)

*Abbreviation*: *ni* not included

Similarly, school-aged children (ages 5–19 years) had a significantly higher risk of *A. lumbricoides* monoinfection, *T. trichiura* monoinfection and *A. lumbricoides* and *T. trichiura* co-infections compared to children aged under 5 years in Luzon, the Visayas, and Mindanao (Tables 2, 3). This was also evident in each infection intensity class for both *A. lumbricoides* and *T. trichiura* infections (Table 4). NDVI was significantly and negatively associated with the prevalence of *A. lumbricoides* and *T. trichiura* co-infections in Mindanao (Table 3). DPWB was significantly and negatively associated with the prevalence of the light infection intensity class for *A. lumbricoides* infection in Mindanao (Table 4).

Semivariograms of model residuals for *A. lumbricoides* monoinfection, *T. trichiura* monoinfection, and co-infections demonstrated spatial autocorrelation in Luzon, the Visayas, and Mindanao (Additional file 1: Figure S1). By contrast, semivariograms of the model residuals demonstrated spatial autocorrelation in Mindanao for all infection intensity classes, except for the prevalence of *T. trichiura* light infection intensity, which indicated no residual spatial autocorrelation (Additional file 1: Figure S2). A detailed summary of parameters of semivariograms for prevalence of mono- and co-infections, and infection intensity classes is provided in Additional file 1: Text S1, Table S3, and Table S4.

### Spatial prediction maps

**Fig. 1:**
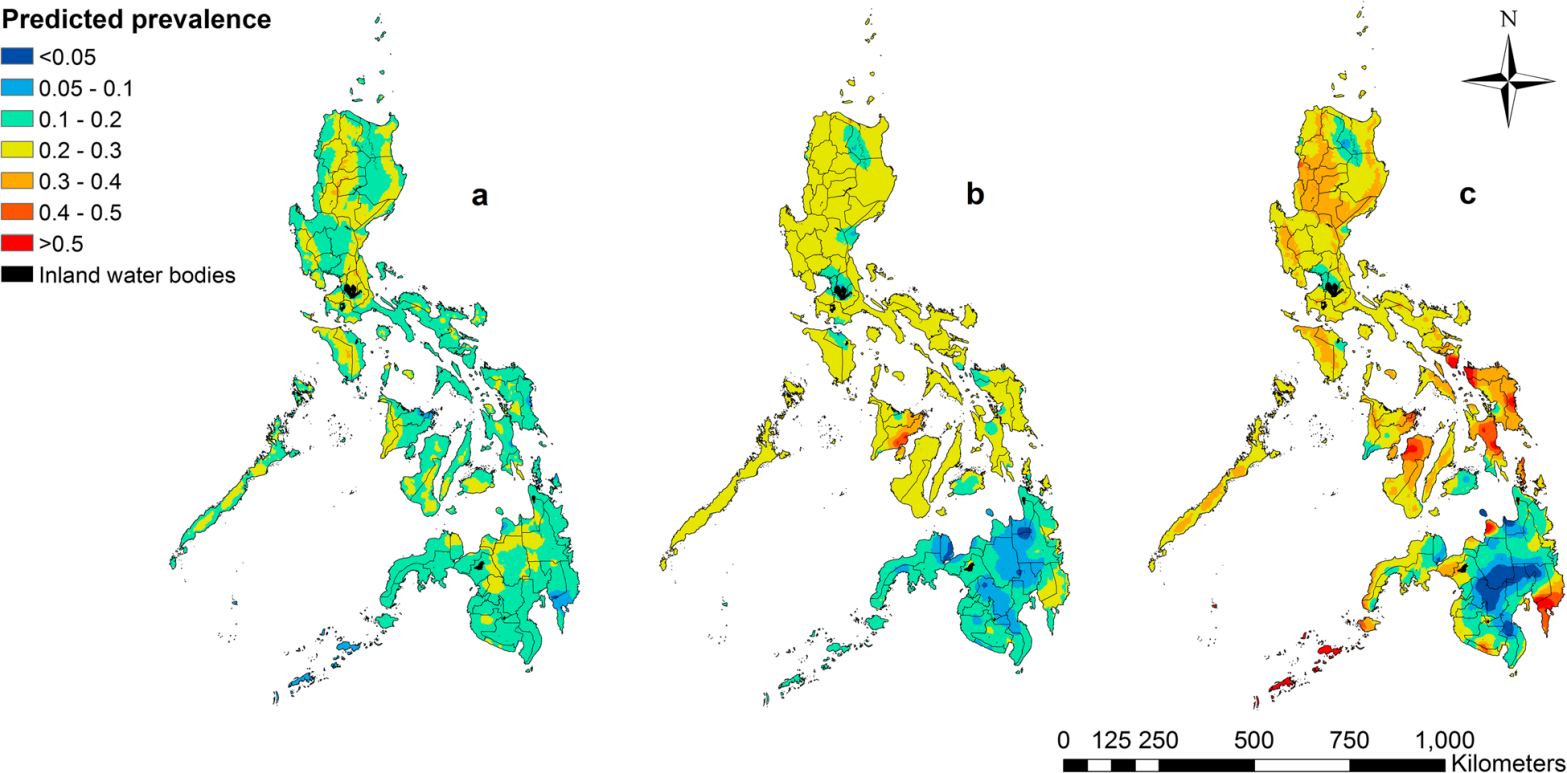
Predicted prevalence of *A. lumbricoides* mono- (**a**), *T. trichiura* mono- (**b**), and co-infections (**c**) in school-aged children in the Philippines. Note: the overall mean predicted prevalences are specific to the age and sex group (choice of a different age-sex group would result in a different spatial mean), with spatial variation around the mean being influenced by the environmental variables and the spatial correlation component of the model

Predicted prevalence of *A. lumbricoides* monoinfection was mostly 10–20% across all regions of the Philippines, with the highest predicted prevalence observed in the north-eastern part of Luzon (> 40%; Fig. 1a). The predicted prevalence of *T. trichiura* monoinfection was mostly 20–30% across Luzon and the Visayas and south-eastern part of Mindanao, with the highest predicted prevalence observed in north-west Visayas (> 40%; Fig. 1b). Predicted prevalence of *A. lumbricoides* and *T. trichiura* co-infections showed a wide distribution across the Philippines, with the highest predicted prevalence observed in the south-eastern tip of Luzon; and eastern division of the Visayas; and the northern and south-eastern divisions of Mindanao, the northeast island province (Sulu) and the southernmost island province (Tawi-Tawi) located in the Autonomous Region in Muslim Mindanao (> 50%; Fig. 1c).

**Fig. 2:**
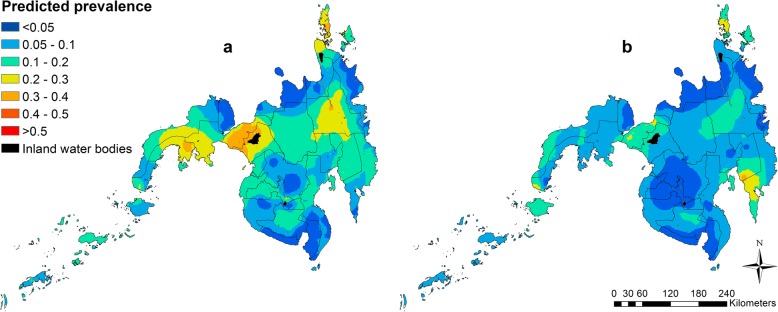
Predicted prevalence of light infection intensity class (**a**) and moderate/high infection intensity classes (**b**) of *A. lumbricoides* in school-aged children in the Philippines

In Mindanao, predicted prevalence of light infection intensity classes for *A. lumbricoides* and *T. trichiura* were predominantly 10–20%, with the highest predicted prevalence of *A. lumbricoides* moderate/high infection intensity observed in the south-eastern part of Mindanao - Davao Oriental and Compostela Valley provinces (> 30%; Fig. 2).

**Fig. 3:**
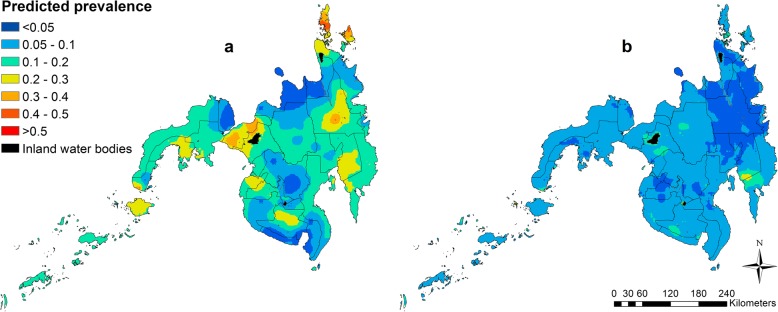
Predicted prevalence of light infection intensity class (**a**) and moderate/high infection intensity classes (**b**) of *T. trichiura* in school-aged children in the Philippines

Similarly, the highest predicted prevalence of *T. trichiura* moderate/high infection was observed in Davao Oriental and Compostela Valley provinces (> 20%; Fig. 3).

Predicted prevalence corresponded well with the observed values (Additional file 1: Figures S3-S7). Predicted standard deviation (SD) of prevalence of *A. lumbricoides* monoinfections, *T. trichiura* monoinfections, co-infections and infection intensity classes are available in Additional file 1: Figures S8-S10.

#### Model validation

The *A. lumbricoides* and *T. trichiura* co-infection model for Mindanao performed satisfactorily (reasonable discriminative ability) for two of the following infection outcomes: *A. lumbricoides* monoinfection (AUC = 0.78 for Mindanao), *T. trichiura* monoinfection (AUC = 0.70 for Mindanao). Discriminatory ability of the co-infection model for *A. lumbricoides* and *T. trichiura* co-infections was just below the threshold (0.69 for Mindanao and 0.68 for Luzon); however, the 95% confidence interval included values of reasonable to good discriminatory ability. Discriminatory ability of *A. lumbricoides* monoinfection, and *T. trichiura* monoinfection for Luzon and the Visayas performed poorly with AUC values below 0.70 (Additional file 1: Table S5). Discriminatory ability of the infection intensity class models for *A. lumbricoides* and *T. trichiura* in Mindanao were poor to reasonable (AUC range 0.52–0.75) (Additional file 1: Table S5).

### Predicted total number of school-aged children with monoinfections, co-infections and infection intensity classes

Areas within and around the capital Manila in Luzon had the highest number of school-aged children with *A. lumbricoides* monoinfection (Additional file 1: Figure S11), with some areas exceeding 45 persons/km^2^. In the Visayas and Mindanao the geographical distribution of the predicted number of school-aged children with *A. lumbricoides* monoinfection was more localised compared to Luzon with some areas exceeding 15 persons/km^2^. Similarly, the number of school-aged children predicted with *T. trichiura* monoinfection (Additional file 1: Figure S12) was distributed widely in the capital Manila in Luzon (some areas with more than 26 persons/km^2^), while the Visayas and Mindanao had small localized areas with more than 23 persons/km^2^ predicted infected. In addition, while in Mindanao the number of school-aged children with *A. lumbricoide*s and *T. trichiura* co-infections (Additional file 1: Figure S13) was predicted to be higher (exceeding 80 persons/km^2^ over a much more localized area around the southernmost tip of Mindanao including Sarangani Province and northern Mindanao including Lanao Del Sur and Misamis Oriental provinces) than in Luzon and the Visayas (27 persons/km^2^and 35 persons/km^2^, respectively), the geographical distribution was more localised in Mindanao compared to Luzon and the Visayas.

In Mindanao, our results showed school-aged children with light or moderate/high infection intensity classes for *A. lumbricoides* were predominantly localised in areas around the southern and northern provinces including Surigao Del Norte and Zamboanga Del Norte (some areas with more than 25 persons/km^2^) (Additional file 1: Figures S14 and S15). School-aged children infected with light or moderate/high infection intensity classes for *T. trichiura* were predicted to be in areas around southern Mindanao and around Lake Buluan near South Cotabato and Davao Del Sur, Maguindanao, Sultan Kudarat (some areas with more than 24 persons/km^2^) (Additional file 1: Figures S16 and S17).

Based on our school-aged children density maps, using the 1 km^2^ grid cells across the Philippines (Additional file 1: Figures S11-S17), our maps of the co-infections identified target provinces where the likelihood of STH-associated morbidity is at its highest, and that spatially targeted integrated helminth control interventions are most needed in Luzon (Bulacan, Benguet, Cavite, Sorsogon, Metropolitan Manila, Pampanga and Rizal) and the central and western islands of the Visayas (Cebu, Iloilo, Leyte and Negros Occidental). Our maps of co-infections and maps of the number of school-aged children with moderate/high infection intensity classes identified target provinces in Mindanao including Agusan Del Norte, Davao Del Sur, Davao Oriental, Lanao Del Sur, Maguindanao, Misamis Occidental, Misamis Oriental, Sulu, Surigao Del Norte, Zamboanga Del Norte, Zamboanga Del Sur and around Lake Buluan.

For 2017, it was estimated that in the Philippines approximately 114,900, 125,200 and 147,900 school-aged children are monoinfected with *A. lumbricoides* and *T. trichiura*, and co-infected with *A. lumbricoides* and *T. trichiura*, respectively. By region, Luzon had the highest estimated number of school-aged children with *A. lumbricoides* and *T. trichiura* co-infections (estimated total 89,400), followed by the Visayas (estimated total 38,300) and Mindanao (estimated total 20,200) (Table 5). In Mindanao, approximately 8300 and 6600 school-aged children were estimated to be infected with moderate/high infection intensity of *A. lumbricoides* and *T. trichiura* infections, respectively, in 2017.

**Table 5:** Predicted number of school-aged children with mono- and co-infection in the Philippines, 2017

Total population for 2015 (millions)^a^	Annual population growth rate for 2015–2020 (%)^b^	Total population for 2017 (millions)^c^	% of individuals aged 5–19 yrs^b^	Region	Number of school-aged children with infection (thousands)^d^						
					Mono- and co-infections			Infection intensity classes			
					Mono-*Asc*	Mono- *Tric*	Co-infection	Light-*Asc*	Moderate/high-*Asc*	Light- *Tric*	Moderate/high-*Tric*
100.7	1.48	103.7	30.2	National	114.9	125.2	147.9	–	–	–	–
				Luzon	68.5	75.7	89.4	–	–	–	–
				The Visayas	22.4	32.7	38.3	–	–	–	–
				Mindanao	24.0	16.8	20.2	15.4	8.3	18.5	6.6

^a^Source: Alpha version 2015 estimates of numbers of people/km^2^, with national totals adjusted to match UN population division estimates (https://esa.un.org/wpp/) and remaining unadjusted; Spatial resolution: 0.000833333 decimal degrees (approx. 100 m at the equator); Projection: Geographic, WGS84; Date of production: November 2013 [34, 42]

^b^Source: The World Population Prospects 2015 Revision Population Database [35, 43]

^c^Estimated value based on the ArcGIS Map algebra raster calculator; 2015 population raster map (the AsiaPop Project) was multiplied by the annual population growth rate for 2015 to 2020

^d^Estimated value based on the ArcGIS Map algebra raster calculator; 2017 population raster map was multiplied by the proportion of the Filipino population aged 5–19 years to derive a map of the number of school-aged children aged 5–19 years in 2017 in each grid cell. We then multiplied the map of the total population aged between 5–19 years by our prediction maps of the prevalence of *A. lumbricoides* monoinfection, *T. trichiura* monoinfection, and *A. lumbricoides* and *T. trichiura* co-infection, and the predicted prevalence of infection intensity classes in ArcGIS software (ESRI 2013. ArcGIS Desktop: Release 10. Redlands, CA: Environmental Systems Research Institute

*Abbreviations*: *Asc A. lumbricoides*; *Tric T. trichiura*

## Discussion

Our modelling approach analysed policy-relevant indicators of STH morbidity and allowed the estimation of the number of school-aged children infected with STH infections in greatest need of benzimidazole treatment in the Philippines. In that regard, our study extends previous spatial epidemiological investigations in the Philippines [13] by predicting the spatial variation in the prevalence of STH co-infections and infection intensity classes in order to identify locations in the Philippines where STH-associated morbidity is at its highest.

### Individual-level determinants of prevalence of STH monoinfection and co-infections, and infection intensity classes

In a recent study, the prevalence of *A. lumbricoides* and *T. trichiura* in the Philippines was estimated to be significantly higher in males compared to females [13]. However, our results showed the reverse relationship for co-infections suggesting that females are more at risk of co-infections in Luzon, the Visayas and Mindanao. Similarly, this was also the case in each infection intensity class for *A. lumbricoides* and *T. trichiura* infections*.* Evidence indicates that the pattern of infections in females is different from that of males due to a number of interacting factors including immunity to infection, response to treatment, and differing levels of exposure related to behaviour and occupational differences [36]. For example, higher prevalence of helminth infections observed in female farmers was reported to be associated with household activities that involved access to open latrines and the manipulation of untreated human faeces in agricultural fields [37]. Further research on the different transmission patterns between males and females is required to encourage the development of gender-sensitive helminth control intervention programmes.

Moreover, consistent with available evidence [9, 20], our results show that school-aged children are at increased risk of *T. trichiura* monoinfection and co-infections compared to children under 5 years in Luzon, the Visayas, and Mindanao. This relationship between age and increased risk of infection was also evident in each infection intensity class for *A. lumbricoides* and *T. trichiura* infections. Our finding on school-aged children being at increased risk of *T. trichiura* monoinfection has implications for future studies to evaluate the type of treatment administered for school-aged children with *T. trichiura* infection. A recent study demonstrated that the most widely used cost-effective preventive chemotherapy with the two benzimidazoles (albendazole and mebendazole) has low to moderate efficacy against *T. trichiura* in high STH endemic countries [38]. Nevertheless, our finding reaffirms the importance of implementing targeted helminth-associated morbidity control for the school-age group.

### The role of environment in the prevalence of monoinfection and co-infections, and STH infection intensity classes

The environmental variables (rainfall, DPWB, LST and NDVI) used in the spatial modelling were found to not adequately explain the spatial variation of *A. lumbricoides* and *T. trichiura* mono- and co-infections based on 95% BCI. While the relative focality of co-infections may be explained by exposure opportunities to contaminated environments [6], the results of this study suggest that small scale factors such as socioeconomic status, occupational exposure, behaviour and access to safe drinking water and to adequate sanitation facilities may be more important than natural environmental variables at explaining the spatial variation in the prevalence of STH co-infections in Luzon and the Visayas [15].

In contrast, in the southernmost region of the Philippines (Mindanao), we found a strong signal in the environmental variables explaining the spatial variation in the prevalence of *A. lumbricoides* and *T. trichiura* co-infections and *A. lumbricoides* light infection intensity class. Our study found that the prevalence of *A. lumbricoides* and *T. trichiura* co-infections was significantly higher in areas of lower greenness (dry areas), which is consistent with the findings of our previous study [13]. This could be due to a number of reasons. For example, in the past couple of decades Mindanao has experienced severe drought [39], which has led to water shortages limiting the access and utilisation of adequate sanitation facilities, contributing to open defecation and inadequate disposal of human waste [40, 41], which in turn is likely to result in a higher prevalence of STH infections [16]. However, NDVI alone cannot fully explain the role of such environmental conditions in the prevalence of *A. lumbricoides* and *T. trichiura* co-infections. Other environmental variables such as type of soil, slope, and normalised difference water index (NDWI) could be added to the spatial modelling to better understand the role of the environment in the prevalence of *A. lumbricoides* and *T. trichiura* mono- and co-infections and infection intensity.

### Populations most in need of interventions to reduce the risk of STH-associated morbidity

Our estimates indicated that for 2017, Luzon had the highest estimated number of school-aged children with *A. lumbricoides* and *T. trichiura* co-infections (estimated total of 89,400 people) followed by the Visayas, (estimated total of 38,300 people) and Mindanao (estimated total of 20,200 people). However, when these estimates were adjusted by population density maps (people/km^2^) we found that areas in Mindanao had the highest density of school-aged children with *A. lumbricoide*s and *T. trichiura* co-infections (82 people/km^2^, compared to 27 people/km^2^ in Luzon and 35 people/km^2^ in the Visayas). This suggests that spatially targeted integrated helminth control interventions in Mindanao may be effective in order to reduce STH-related morbidity. Maps of infection intensity classes for Mindanao indicated that the number of school-aged children predicted to be infected with light or moderate/high infection intensity classes for *A. lumbricoides* and *T. trichiura* were highest in areas around the northern provinces and southern Mindanao (some areas with more than 24 persons/km^2^). Together with the predicted number of co-infections these results suggest that areas of priority in Mindanao would include Agusan Del Norte, Davao Del Sur, Davao Oriental, Lanao Del Sur, Maguindanao, Misamis Occidental, Misamis Oriental, Sulu, Surigao Del Norte, Zamboanga Del Norte, Zamboanga Del Sur and around Lake Buluan.

In addition, spatial variability of the prevalence of co-infection across the Philippines is suggestive of differing impacts of anthropogenic and natural environmental factors. For instance, many suburbs around the capital city of Manila in Luzon are densely populated areas (except the Sorsogon Province which is known for fisheries), which means urbanization (including establishment of slums), overcrowding and sanitation could be influencing the prevalence of STH infections among school-aged children in this region [29]. However, provinces in the Visayas and Mindanao are mostly rural areas known for agriculture and fisheries, which means that school-aged children in these areas may have more contact with the natural environment and agricultural work compared to those living in Luzon.

### Limitations of the study

The results of the study should be interpreted in the light of some limitations. First, our study used secondary data collected in 2005–2007 and may not fully reflect the current situation. However, these data constitute the most up to date information on the prevalence of STH infections in the Philippines. The most recent studies only cover the prevalence of STH infections on a regional level [15, 18, 24, 25], but provide sufficient evidence to suggest that the national level of prevalence is still similar to that of the 2005–2007 survey. Therefore, we anticipate our survey data is still relevant. Secondly, parasite egg counts were based on a single sample which limits the Kato-Katz test performance for detecting light infection intensities [4, 18]. Therefore, the results reported in our study may have underestimated the prevalence of STH infections. The accuracy of estimates of the association between covariates and STH infection could be improved by adjusting for potential measurement error in eggs per gram of faeces (epg) counts. Thirdly, while this study was able to produce prediction maps of *A. lumbricoides* and *T. trichiura* co-infections for all three regions, we did not have enough information on infection intensity classes for the Visayas and Luzon. Further studies or surveys are suggested especially targeting the areas predicted to have the high prevalence of co-infections in the Visayas and Luzon. This approach would allow estimation of the number of school-aged children with co-infections and high infection intensity who are at increased risk of severe morbidity, and are most in need of interventions. Fourthly, while the 95% confidence interval of validation statistics (AUC) included values of acceptable discriminatory ability for all models, the discriminatory outcomes of co-infections for Luzon and the Visayas, and Mindanao moderate/high infection intensity classes’ models were below the threshold. This may be due to our data on these infections being sparse for these regions and thus additional surveys would be required to improve the accuracy of our prediction models. Fifthly, small scale factors such as socioeconomic status, occupational exposure, behaviour, household or water sanitation factors are also known to be important determinants of the prevalence of STH infections. Unfortunately, data on these factors were not available. This was however was captured by residuals, thus we are accounting for that by including spatial random effects in our models.

## Conclusions

Overall, our study generated an important epidemiological resource, highlighting national priority areas for STH morbidity control in the Philippines. Our findings can guide policy makers in the Philippines to design geographically targeted intervention programmes to reduce STH-associated morbidity. This is particularly important in order for the Philippines to achieve the target of less than 20% prevalence of STH infections, and zero prevalence of moderate and high intensity STH infections recommended by the WHO to achieve morbidity control.

## Additional file

Additional file 1: **Text S1.** Analysis of residual spatial dependence. **Text S2.** Model specification. **Table S1.** Number of individuals with two or more STH infections. **Table S2.** Number of individuals with mono- and co-infections. **Table S3.** Summary of parameters of semivariograms for prevalence of mono- and co-infections. **Table S4.** Summary of parameters of semivariograms for prevalence of infection intensity classes. **Table S5.** Summary of validation statistics for models used in study. **Figure S1.** Semivariograms of prevalence of co-infection models. **Figure S2.** Semivariograms of prevalence of infection intensity classes model. **Figure S3.** Map of observed prevalence of mono- and co-infections in Luzon. **Figure S4.** Map of observed prevalence of mono- and co-infections in the Visayas. **Figure S5.** Map of observed prevalence of mono- and co-infections in Mindanao. **Figure S6.** Map of observed infection intensity classes of *A. lumbricoides* in Mindanao. **Figure S7.** Map of observed infection intensity classes of *T. trichiura* in Mindanao. **Figure S8.** Maps of standard deviation of predicted prevalence of *A. lumbricoides* mono-, *T. trichiura* mono-, and co-infection. **Figure S9.** Maps of standard deviation of predicted prevalence of infection intensity classes of *A. lumbricoides*. **Figure S10.** Maps of standard deviation of predicted prevalence of infection intensity classes of *T. trichiura*. **Figure S11.** Maps showing number of individuals with *A. lumbricoides* monoinfection. **Figure S12.** Maps showing number of individuals with *T. trichiura* monoinfection. **Figure S13.** Maps showing number of individuals with co-infections. **Figure S14.** Map showing number of individuals with light intensity class of *A. lumbricoides*. **Figure S15.** Map showing number of individuals with moderate/high intensity classes of *A. lumbricoides*. **Figure S16.** Map showing number of individuals with light intensity class of *T. trichiura*. **Figure S17.** Map showing number of individuals with moderate/high intensity classes of *T. trichiura*. (DOCX 4419 kb)

## Abbreviations

AIC: Akaike’s information criterion
AUC: Area under the curve
BCI: Bayesian credible interval
DALYs: Disability-adjusted life years
DPWB: Distance to perennial water bodies
EPG: Eggs per gram of faeces
GIS: Geographical Information System
LST: Land surface temperature
MDA: Mass drug administration
NOAA: National Oceanographic and Atmospheric Administrations
NDVI: Normalized difference vegetation index
SD: Standard deviation
STH: Soil-transmitted helminths
UNDP: United Nations Development Programme
WHO: World Health Organization
YLDs: Years lived with disability
