# Supplementary material for: Spatial distribution and populations at risk of A. lumbricoides and T. trichiura co-infections and infection intensity classes: an ecological study
Source: Parasit Vectors. 2018 Oct 3;11:535. doi: 10.1186/s13071-018-3107-y (PMC6171148; doi:10.1186/s13071-018-3107-y)
Supplement: Supplementary file 1 — Text S1. Analysis of residual spatial dependence. Text S2. Model specification. Table S1. Number of individuals with two or more STH infections. Table S2. Number of individuals with mono- and co-infections. Table S3. Summary of parameters of semivariograms for prevalence of mono- and co-infections. Table S4. Summary of parameters of semivariograms for prevalence of infection intensity classes. Table S5. Summary of validation statistics for models used in study. Figure S1. Semivariograms of prevalence of co-infection models. Figure S2. Semivariograms of prevalence of infection intensity classes model. Figure S3. Map of observed prevalence of mono- and co-infections in Luzon. Figure S4. Map of observed prevalence of mono- and co-infections in the Visayas. Figure S5. Map of observed prevalence of mono- and co-infections in Mindanao. Figure S6. Map of observed infection intensity classes of A. lumbricoides in Mindanao. Figure S7. Map of observed infection intensity classes of T. trichiura in Mindanao. Figure S8. Maps of standard deviation of predicted prevalence of A. lumbricoides mono-, T. trichiura mono-, and co-infection. Figure S9. Maps of standard deviation of predicted prevalence of infection intensity classes of A. lumbricoides. Figure S10. Maps of standard deviation of predicted prevalence of infection intensity classes of T. trichiura. Figure S11. Maps showing number of individuals with A. lumbricoides monoinfection. Figure S12. Maps showing number of individuals with T. trichiura monoinfection. Figure S13. Maps showing number of individuals with co-infections. Figure S14. Map showing number of individuals with light intensity class of A. lumbricoides. Figure S15. Map showing number of individuals with moderate/high intensity classes of A. lumbricoides. Figure S16. Map showing number of individuals with light intensity class of T. trichiura. Figure S17. Map showing number of individuals with moderate/high intensity classes of T. trichiura. (DOCX 4419 kb) [file 13071_2018_3107_MOESM1_ESM.docx]

**Additional file 1**

**Text S1**

Analysis of residual spatial dependence. A semivariogram is a graphical representation of the spatial variation in a dataset; residual semivariograms represent the spatial variation left unexplained after the inclusion of covariates in a model. Omnidirectional semivariograms were generated for *A. lumbricoides* monoinfection, *T. trichiura* monoinfection, and *A. lumbricoides* and *T. trichiura* co-infections; and for each level of infection intensity for *A. lumbricoides* and *T. trichiura* species. This was done for each region of the Philippines. The semivariogram is characterised by three parameters: the partial sill, which is the spatially structured component of the semivariance (indicative of the tendency for geographical clustering), the nugget, which is the spatially unstructured component of the semivariance (representing random variation, very small-scale spatial variability or measurement error) and the range, which is the distance at which locations can be considered independent (indicative of the average size of geographical clusters) [[1](#_ENREF_1)]. The tendency for geographical clustering within a region (i.e. proportion of variation that is due to spatial proximity) was estimated by dividing the partial sill by the sum of the nugget and the partial sill.

The estimated cluster size for *A. lumbricoides* and *T. trichiura* co-infections were larger compared to *A. lumbricoides* monoinfection and *T. trichiura* monoinfection in Luzon and the Visayas (See Additional file 1: Table S3). While similar cluster size was observed for the prevalence of moderate/high infection intensity for *A. lumbricoides* and *T. trichiura* infections, the largest estimated cluster size was for the prevalence of moderate/high infection intensity for *T. trichiura* infections (See Additional file 1: Table S4).

Our study, together with the results of the semivariograms, indicates that strategies for helminth control programs in school-aged children in the Philippines need to consider local conditions, taking into account spatially-dependent determinants of the prevalence of STH infection.

**Text S2**

**Model specification.** Bayesian model outputs for parameters of interest and for predictions at unsampled locations are probability distributions, termed posterior distributions, which represent the probability of a variable of interest taking each of a range of plausible values [[1](#_ENREF_1)]. The posterior distributions can be summarized by statistics such as the posterior mean and 95% Bayesian credible interval (BCI). For model coefficients, a variable was considered as influencing the outcome if it excluded zero. We use marginal prediction using *spatial.unipred* command, which implements independent simulations that do not consider neighbouring values. For each model, a burn-in of 35,000 Markov Chain Monte Carlo iterations was used followed by 5,000 iterations during which values for the intercept and coefficients were stored for parameter estimation and generation of predictive maps. Diagnostic tests for convergence of the stored variables were assessed using visualization of history and density plots of the series of posterior values.

**References**

1. Soares Magalhaes RJ, Clements AC, Patil AP, Gething PW, Brooker S. The applications of model-based geostatistics in helminth epidemiology and control. Adv Parasitol. 2011;74:267-96.

**Tables**

**Table S1.** Number of individuals with two or more STH infections, all age groups included.

| Infection with two or more STH infections | Luzon and the Visayas (N=10276) | Mindanao (N=19643) |
| --- | --- | --- |
| *T. trichiura* / Hookworm (%) | 460 (4.5) | 465 (2.4) |
| *A. lumbricoides* / Hookworm (%) | 383 (3.7) | 454 (2.3) |
| *A. lumbricoides* / *T. trichiura* (%) | 2223 (21.6) | 1647 (8.4) |
| *A. lumbricoides* / *T. trichiura* / Hookworm (%) | 312 (3.0) | 175 (0.9) |

**Table S2.** Number of individuals with mono- and co-infections, stratified by sex and age groups.

| Region | Total | | Sex | | | | Age group (age in years) | | | | | | |
| --- | --- | --- | --- | --- | --- | --- | --- | --- | --- | --- | --- | --- | --- |
|  |  |  | Male | | Female | | Under 5 | | 5 to 19 | | 20 and older | | |
|  | L&V | Min | L&V | Min | L&V | Min | L&V | Min | L&V | Min | L&V | Min |  |
| Total number of individuals | 10276 | 19643 | 4861 | 9586 | 5415 | 10057 | 1241 | 2017 | 4037 | 8080 | 4998 | 9546 |  |
| *A. lumbricoides* monoinfection (%) | 1041  (10.2) | 2507  (12.7) | 465  (44.7) | 1237  (49.3) | 576  (55.3) | 1270  (50.7) | 203  (16.4) | 381  (18.9) | 411  (10.2) | 1206  (14.9) | 427  (8.6) | 920  (9.6) |  |
| *T. trichiura* monoinfection (%) | 1885  (18.3) | 1664  (8.5) | 926  (49.1) | 818  (49.2) | 959  (50.9) | 846  (50.8) | 144  (11.6) | 105  (5.2) | 780  (19.3) | 773  (9.6) | 961  (19.2) | 786  (8.2) |  |
| *A. lumbricoides* and *T. trichiura* co-infections (%) | 2223  (21.6) | 1647  (8.4) | 1075  (48.4) | 781  (47.4) | 1148  (51.6) | 866  (52.6) | 257  (20.7) | 174  (8.6) | 1155  (28.6) | 908  (11.2) | 811  (16.2) | 565  (5.9) |  |

*Abbreviations: L&V = Luzon and the Visayas; Min = Mindanao*.**Table S3.** Summary of parameters of semivariograms for prevalence of mono- and co-infections.

| Regions / Parameters | *A. lumbricoides*-mono | *T. trichiura*-mono | *A. lumbricoides* and *T. trichiura* co-infections |
| --- | --- | --- | --- |
| Luzon and the Visayas |  |  |  |
| Partial sill | 0.0049 | 0.0010 | 0.0005 |
| Nugget | 0.0054 | 0.0004 | 0.0001 |
| Practical range | 1.775 | 1.797 | 2.097 |
| % of the variance due to clustering | 47.74 | 71.43 | 84.75 |
| Mindanao |  |  |  |
| Partial sill | 0.0019 | 0.0001 | 0.0001 |
| Nugget | 0.0004 | 4.00E-05 | 1.00E-5 |
| Practical range | 1.495 | 1.498 | 0.899 |
| % of the variance due to clustering | 82.61 | 71.43 | 92.86 |

**Table S4.** Summary of parameters of semivariograms for prevalence of infection intensity classes.

| Region / Parameters | *A. lumbricoides* | | *T. trichiura* | |
| --- | --- | --- | --- | --- |
| Mindanao | Light | Moderate/high | Light | Moderate/high |
| Partial sill | 0.0014 | 0.0001 | 0.0001 | 0.0001 |
| Nugget | 0.0001 | 0.00E+00 | 4.00E-05 | 0.00E+00 |
| Practical range | 1.498 | 1.518 | 1.513 | 1.531 |
| % of the variance due to clustering | 95.79 | 100.00 | 71.43 | 100.00 |

**Table S5.** Summary of validation statistics for models used in study.

| Regions / Models | Area under the ROC ^a^ curve | Standard error | 95% CI ^b^ |
| --- | --- | --- | --- |
| Prevalence of *A. lumbricoides* and *T. trichiura* mono- and co-infections | | | |
| Luzon and the Visayas |  |  |  |
| *A. lumbricoides* monoinfection | 0.58 | 0.15 | 0.29, 0.86 |
| *T. trichiura* monoinfection | 0.56 | 0.14 | 0.28, 0.83 |
| Co-infections | 0.68 | 0.13 | 0.43, 0.92 |
| Mindanao |  |  |  |
| *A. lumbricoides* monoinfection | 0.78 | 0.09 | 0.58, 0.97 |
| *T. trichiura* monoinfection | 0.70 | 0.14 | 0.43, 0.97 |
| Co-infections | 0.69 | 0.14 | 0.42, 0.95 |
| Prevalence of infection intensity classes (Mindanao only) | | | |
| Light *A. lumbricoides* | 0.72 | 0.13 | 0.47, 0.97 |
| Moderate/high *A. lumbricoides* | 0.65 | 0.13 | 0.40, 0.89 |
| Light *T. trichiura* | 0.75 | 0.11 | 0.53, 0.96 |
| Moderate/high *T. trichiura* | 0.52 | 0.13 | 0.27, 0.77 |

**Figures**

| Region | *A. lumbricoides* monoinfection | *T. trichiura* monoinfection | *A. lumbricoides* and *T. trichiura* co-infection |
| --- | --- | --- | --- |
| Luzon and the Visayas | 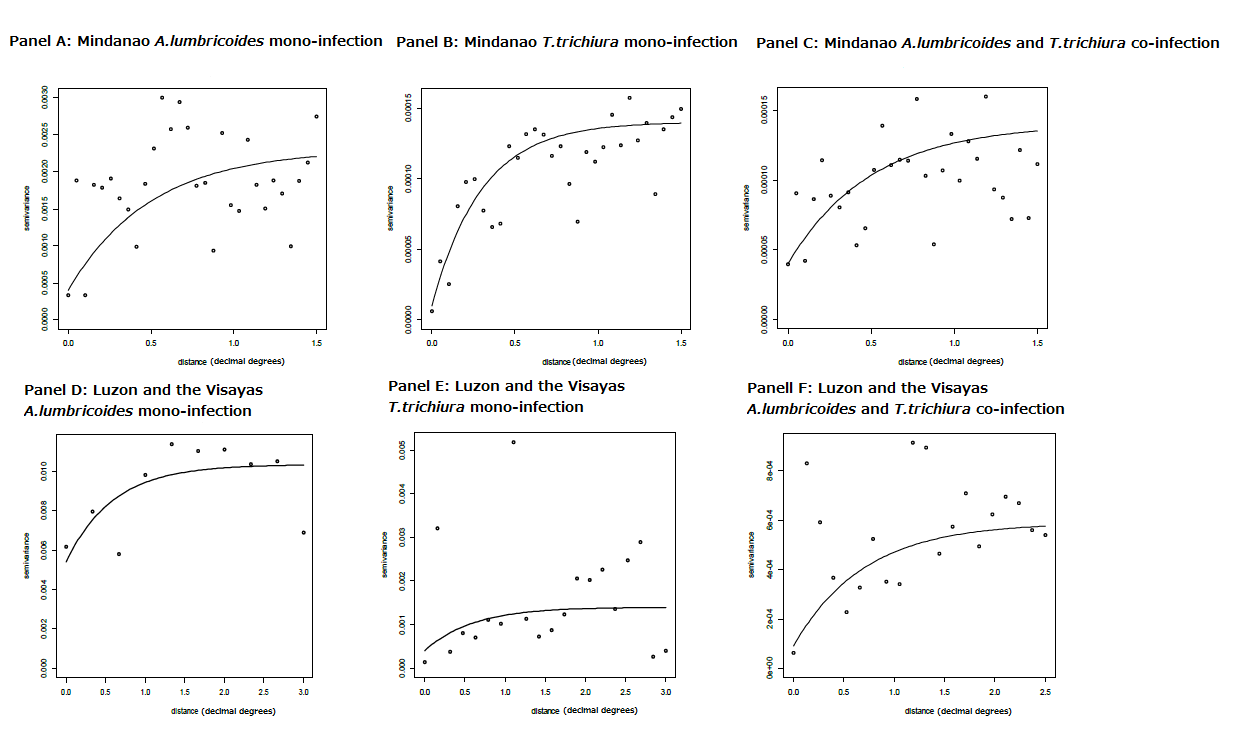 | 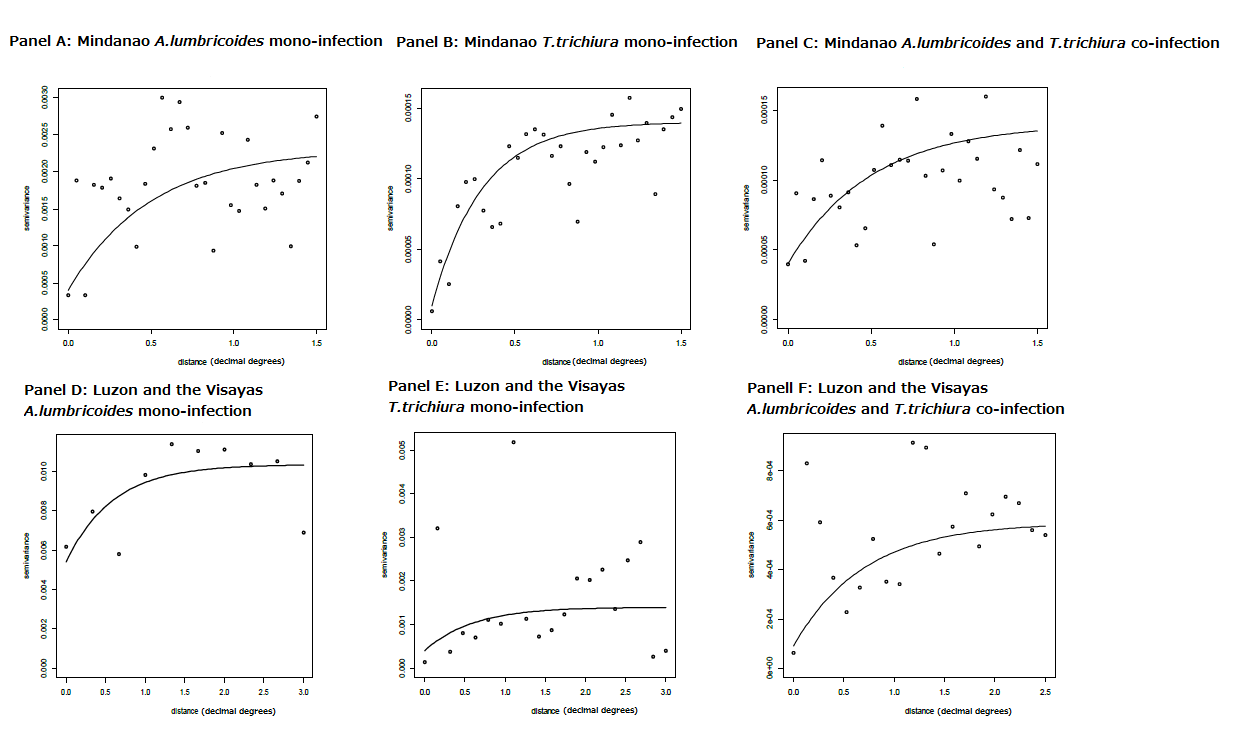 | 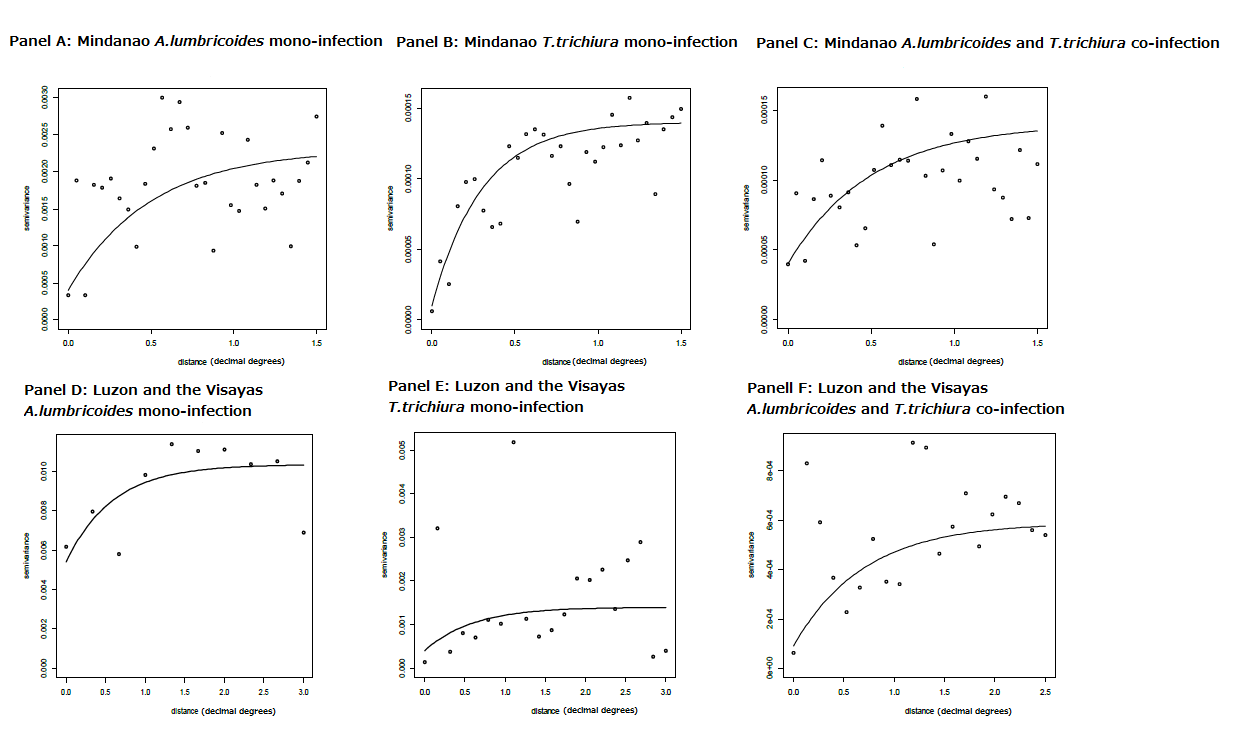 |
| Mindanao | 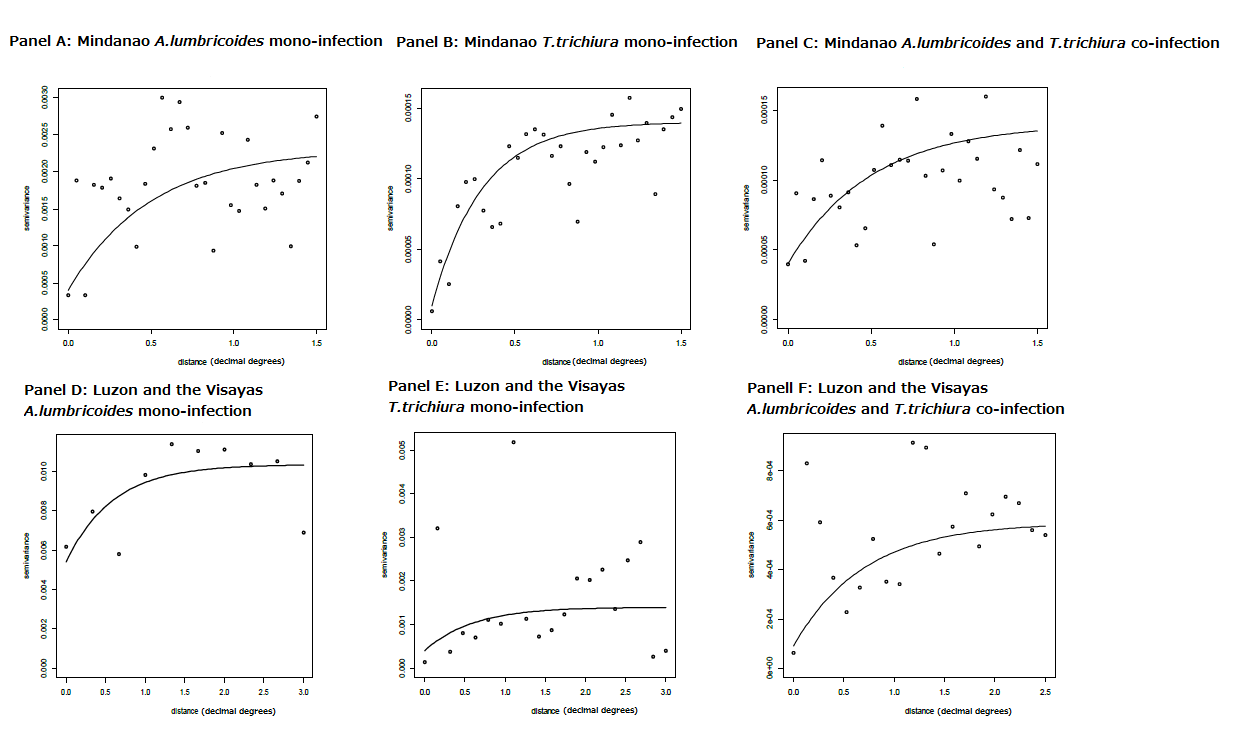 | 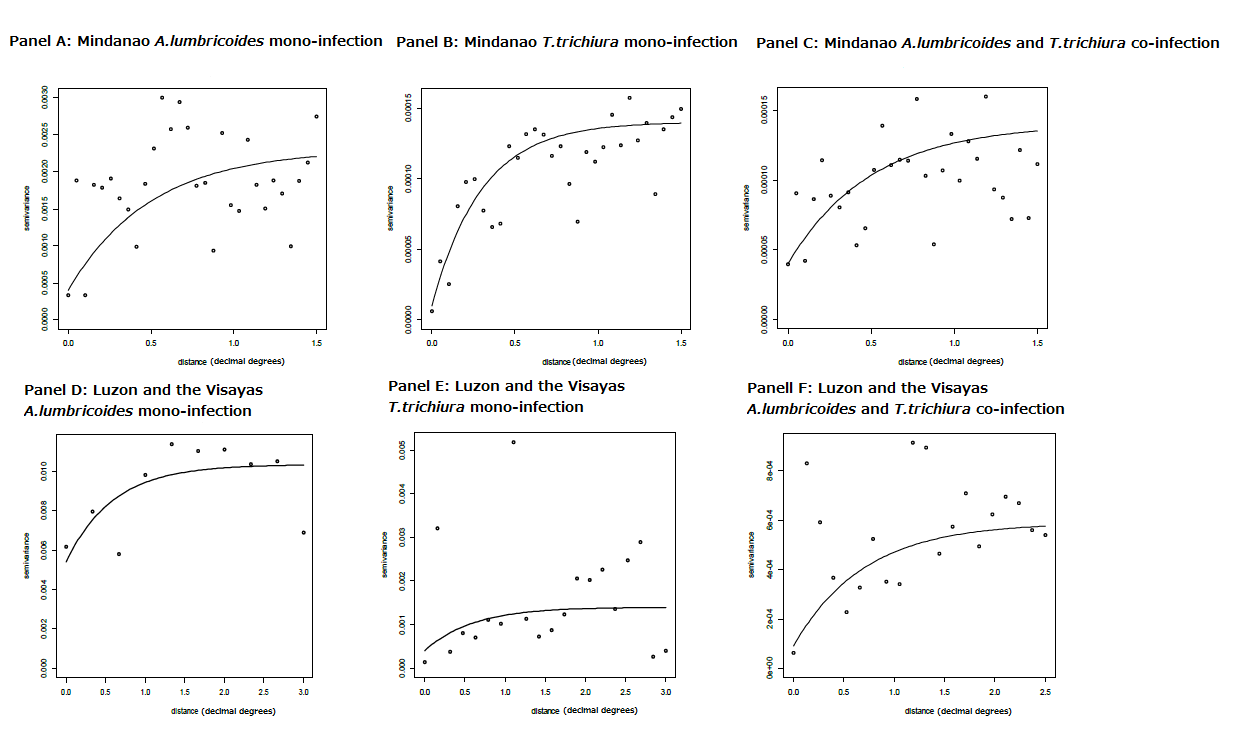 | 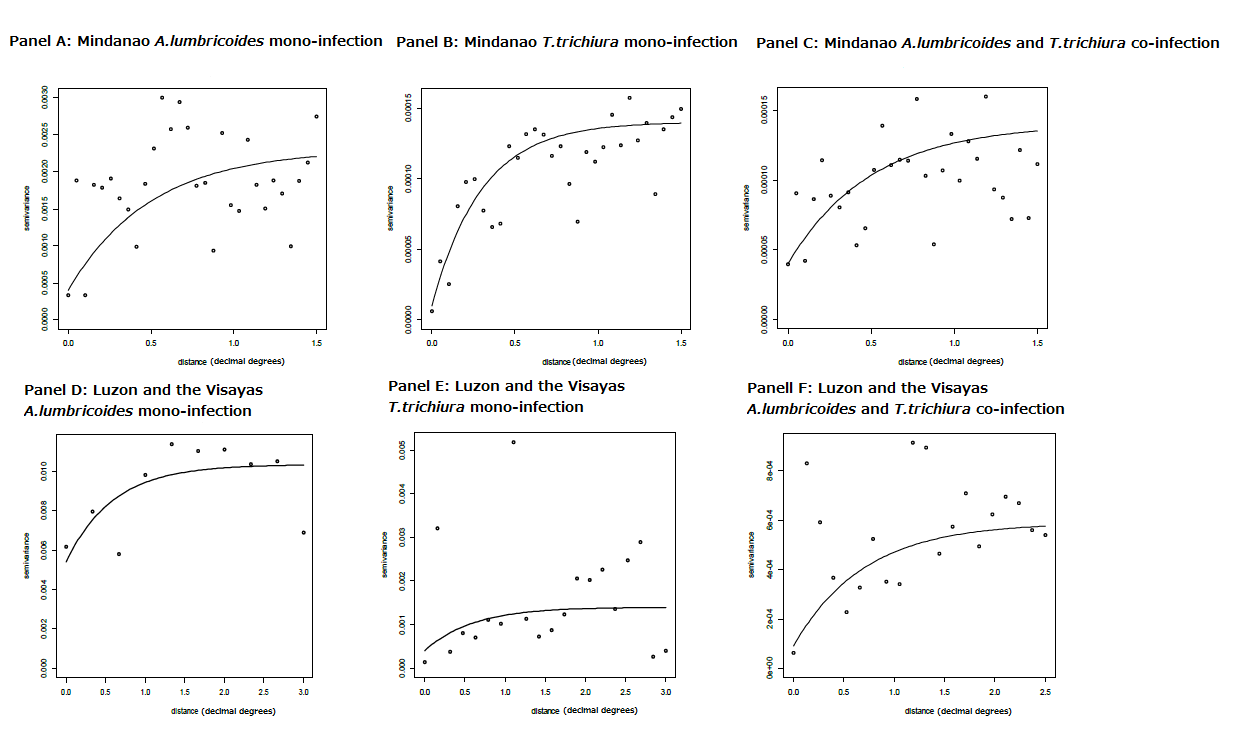 |

**Figure S1.** Semivariograms of residual spatial dependence, the prevalence of mono- and co-infection models.

| STH | Light infection intensity class | Moderate/high intensity classes |
| --- | --- | --- |
| *A. lumbricoides* | 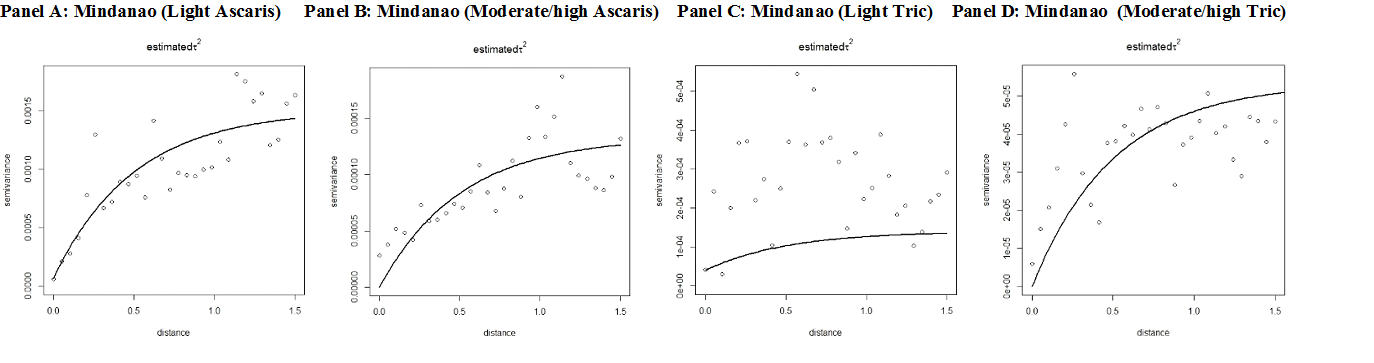 | 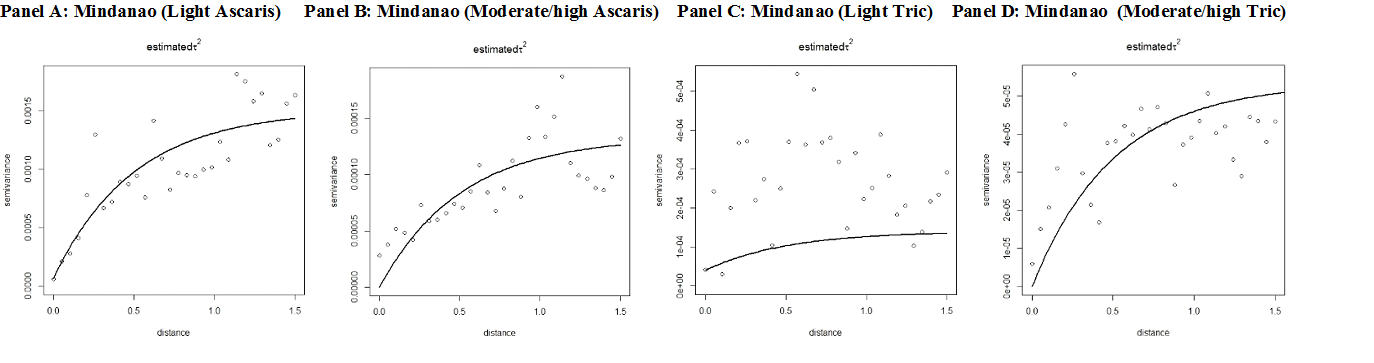 |
| *T. trichiura* | 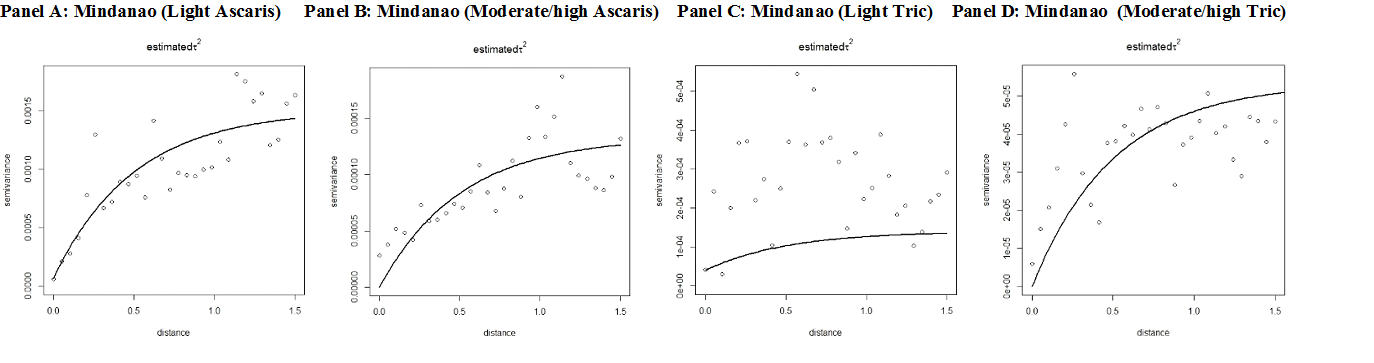 | 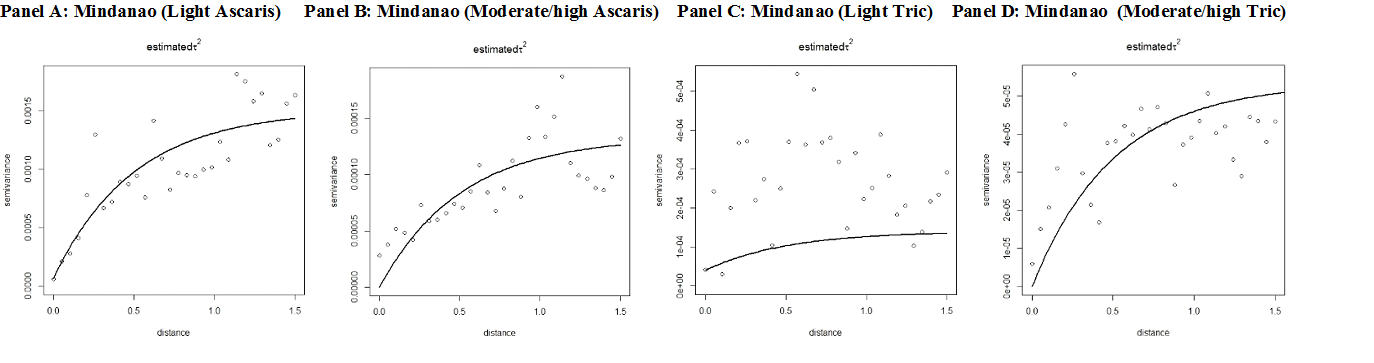 |

**Figure S2.** Semivariograms of residual spatial dependence, the prevalence of *A. lumbricoides* and *T. trichiura* infection intensity classes model.


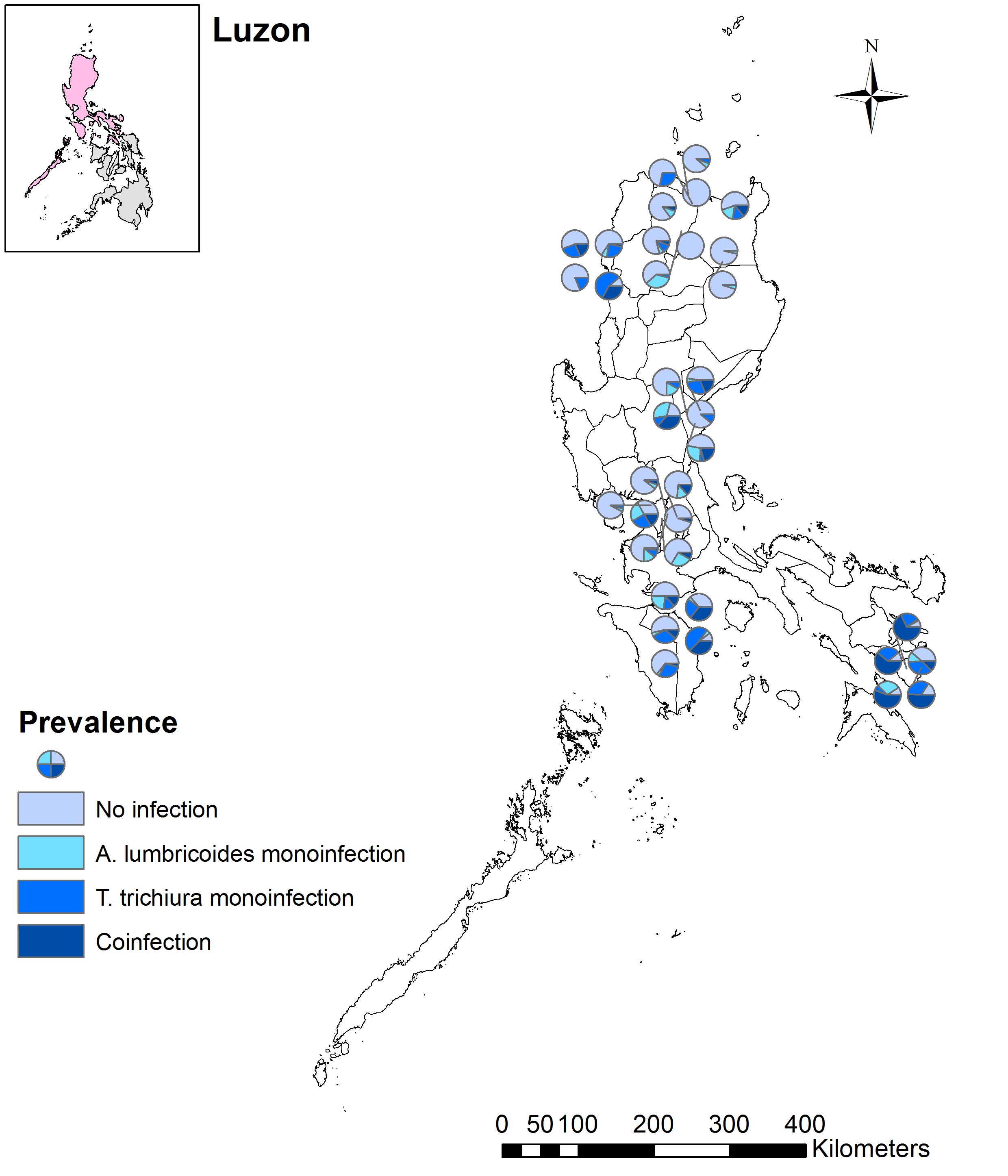


**Figure S3.** Map of observed prevalence of *A. lumbricoides* and *T. trichiura* mono- and co-infections in school-aged children in Luzon, 2005 – 2007.


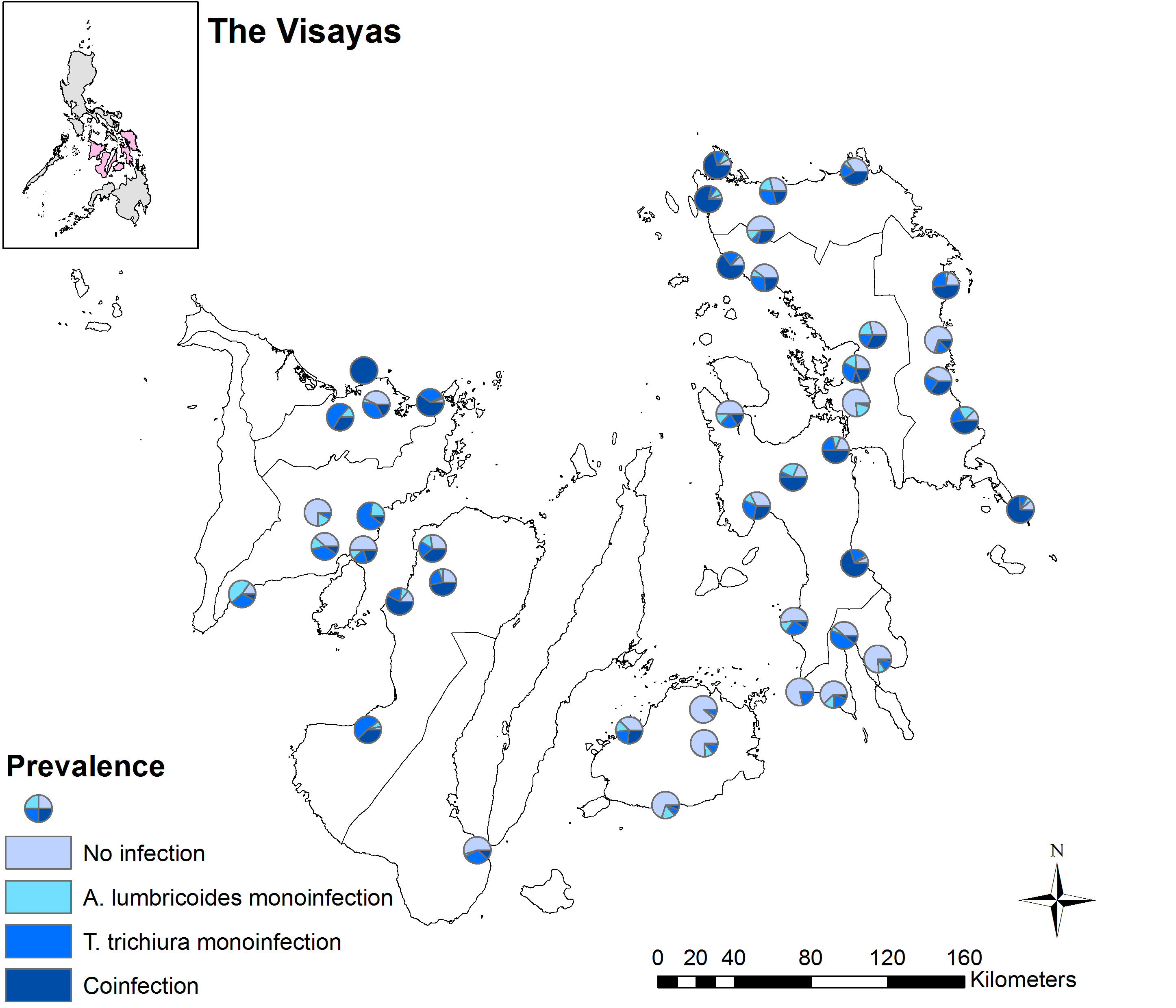


**Figure S4.** Map of observed prevalence of *A. lumbricoides* and *T. trichiura* mono- and co-infections in school-aged children in the Visayas, 2005 – 2007.


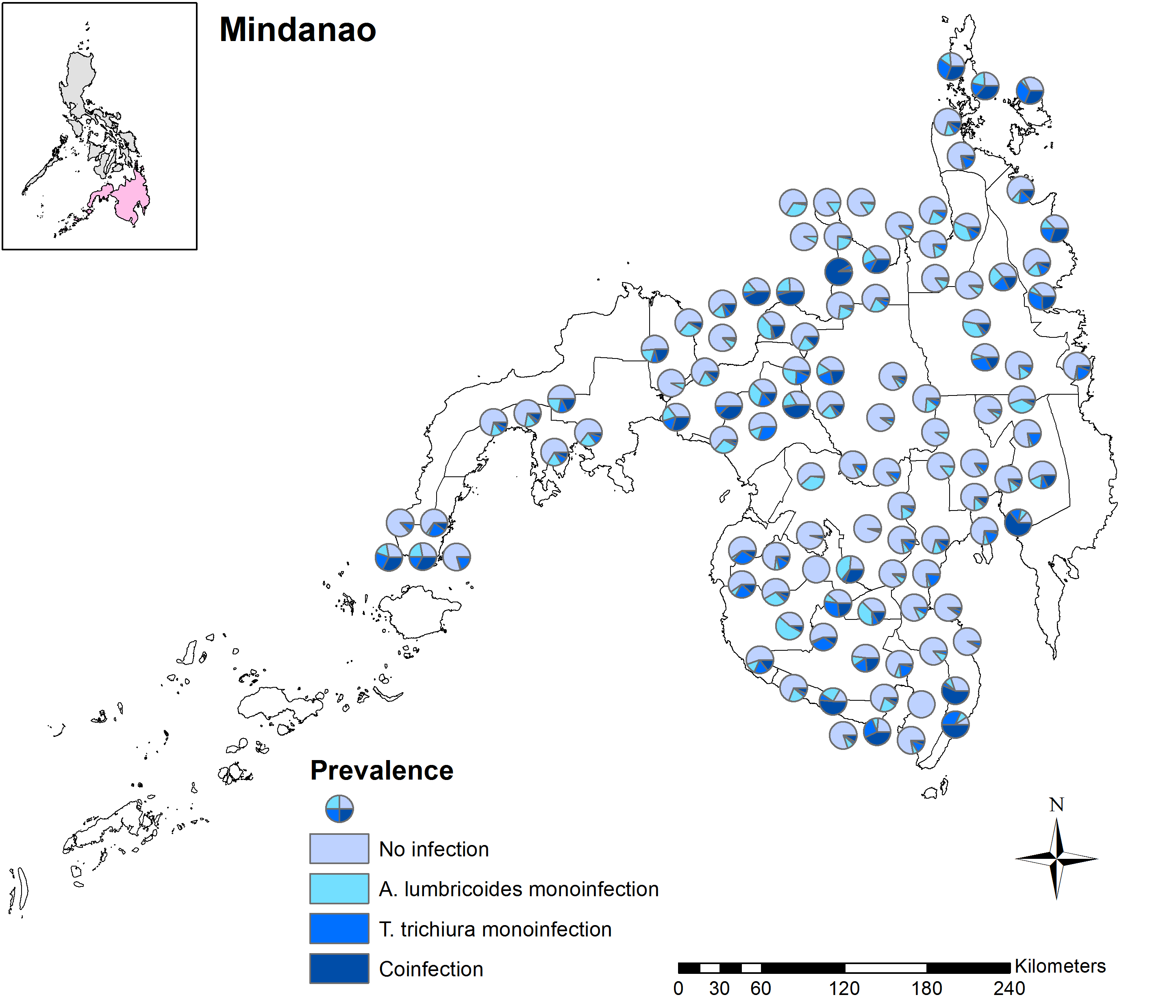


**Figure S5.** Map of observed prevalence of *A. lumbricoides* and *T. trichiura* mono- and co-infections in school-aged children in Mindanao, 2005 – 2007.
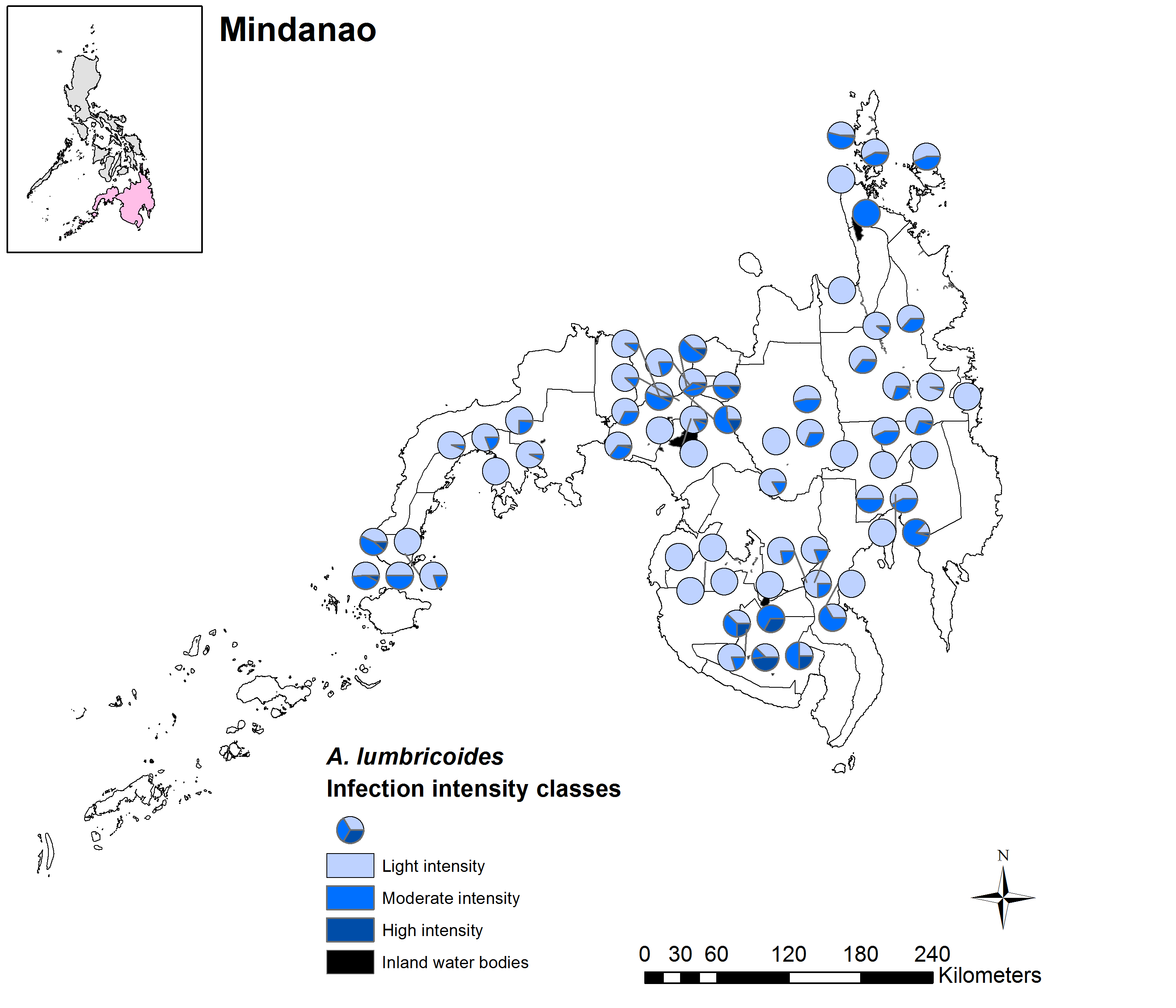
**Figure S6.** Map of observed infection intensity classes of *A. lumbricoides* in school-aged children in Mindanao, 2005 – 2007.


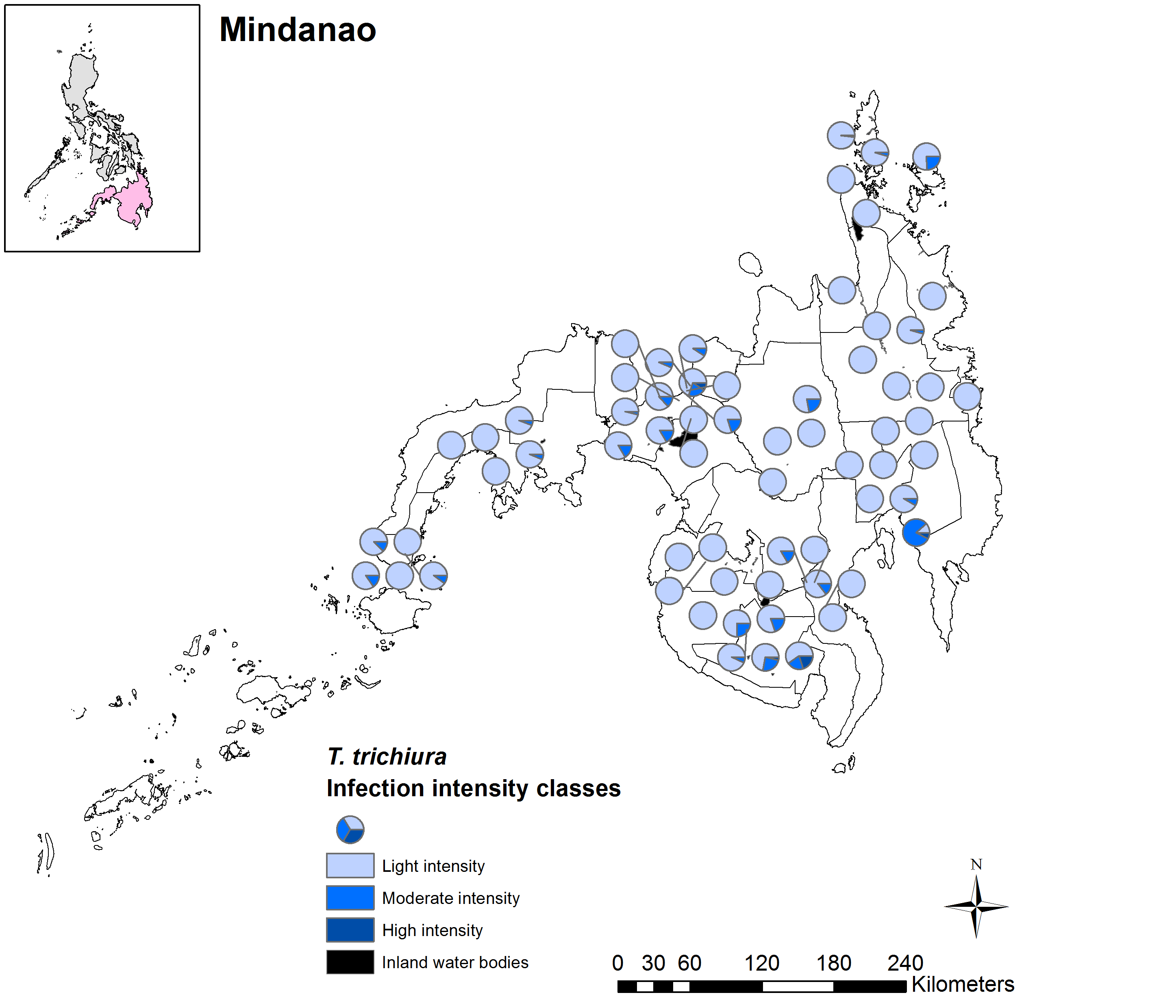


**Figure S7.** Map of observed infection intensity classes of *T. trichiura* in school-aged children in Mindanao, 2005 – 2007.

| **a**  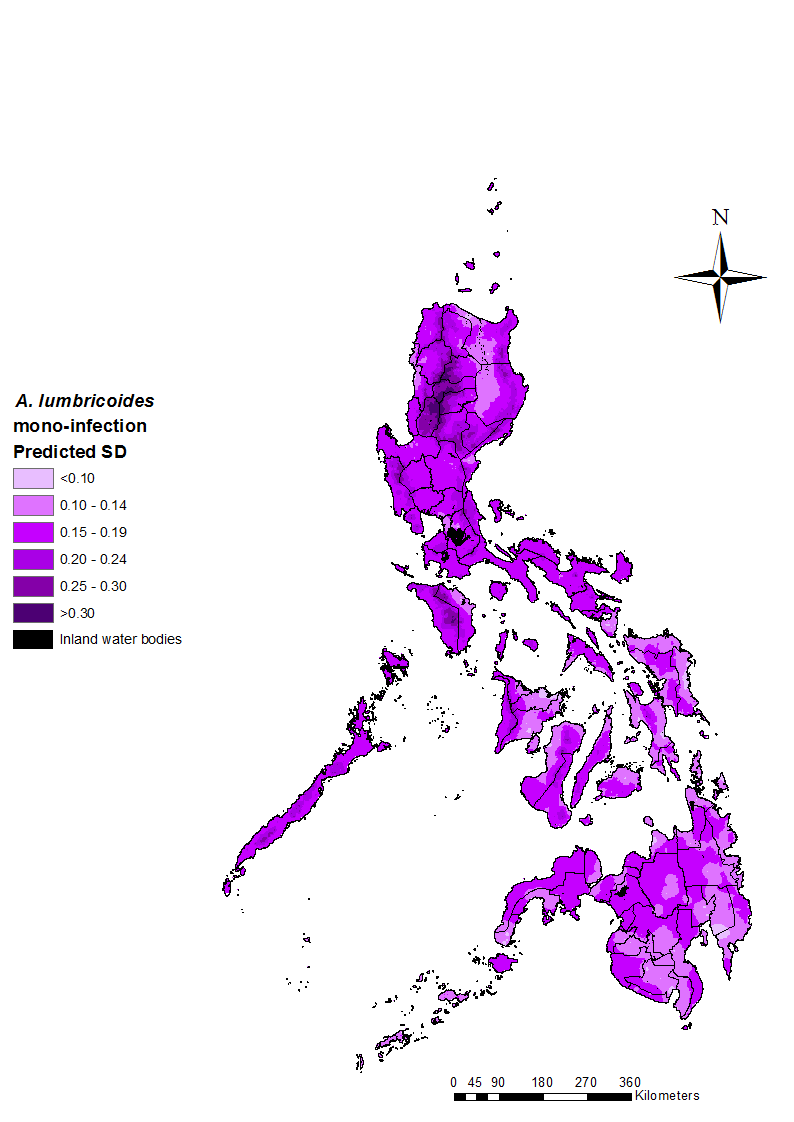 | **b**  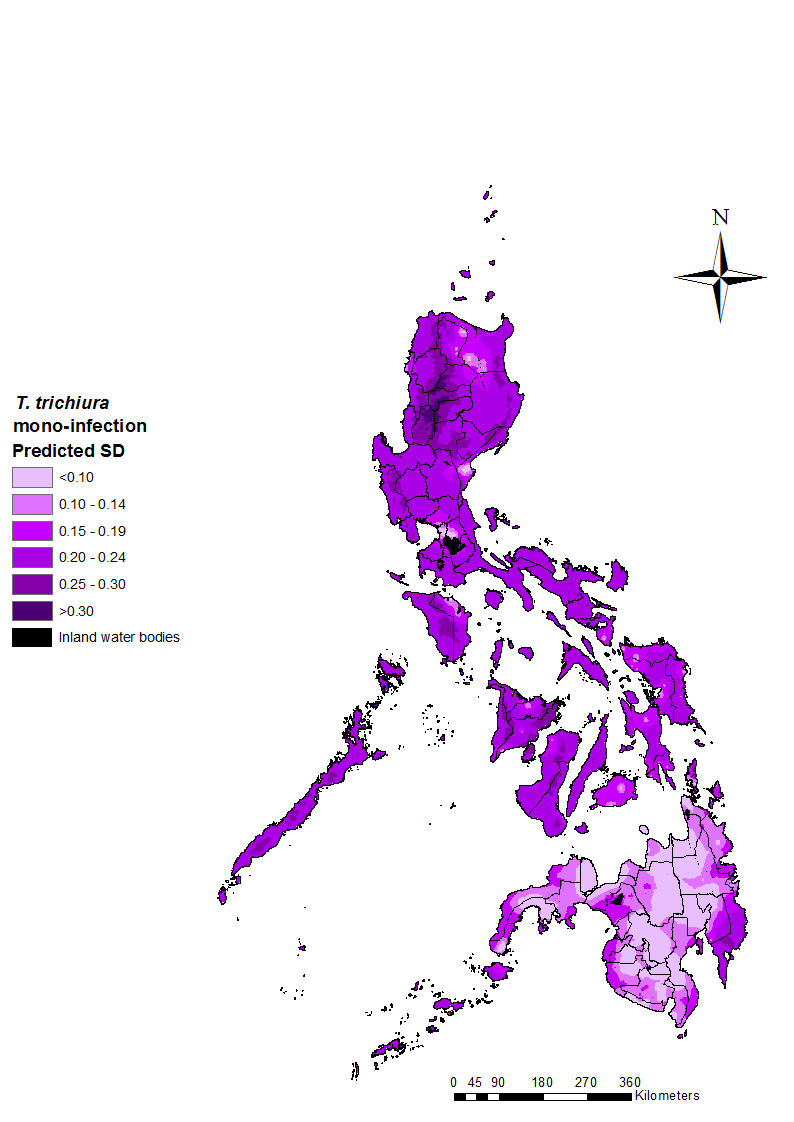 | **c**  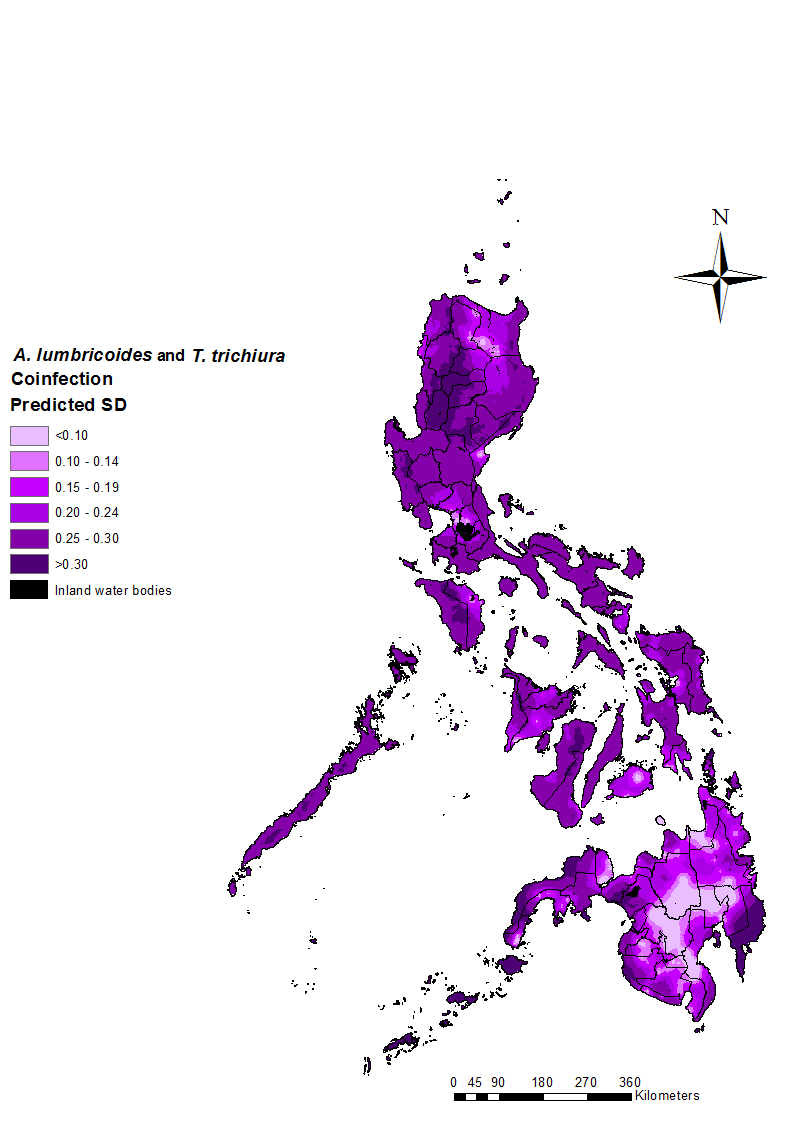 |
| --- | --- | --- |

**Figure S8.** Maps of standard deviation (SD) of predicted prevalence of *A. lumbricoides* mono- (**a**), *T. trichiura* mono- (**b**), and co-infection (**c**) in school-aged children in the Philippines, 2017.

**a**


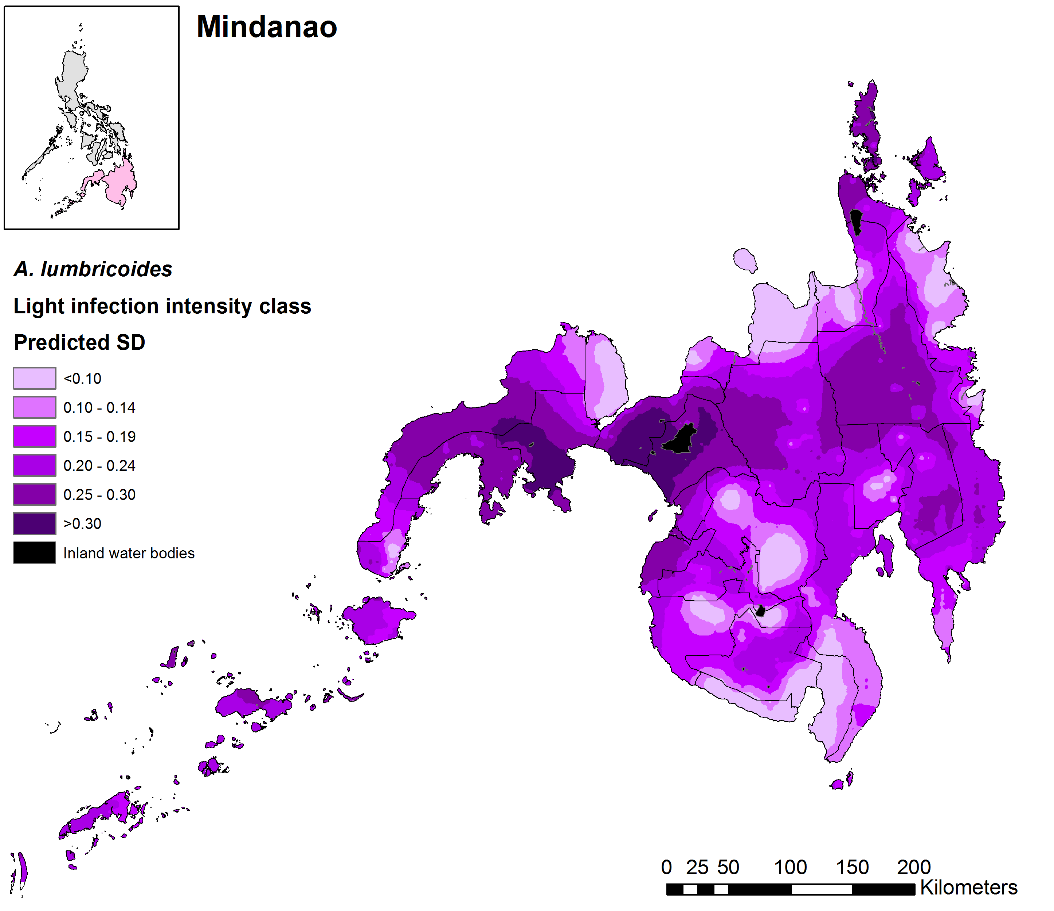


**b**


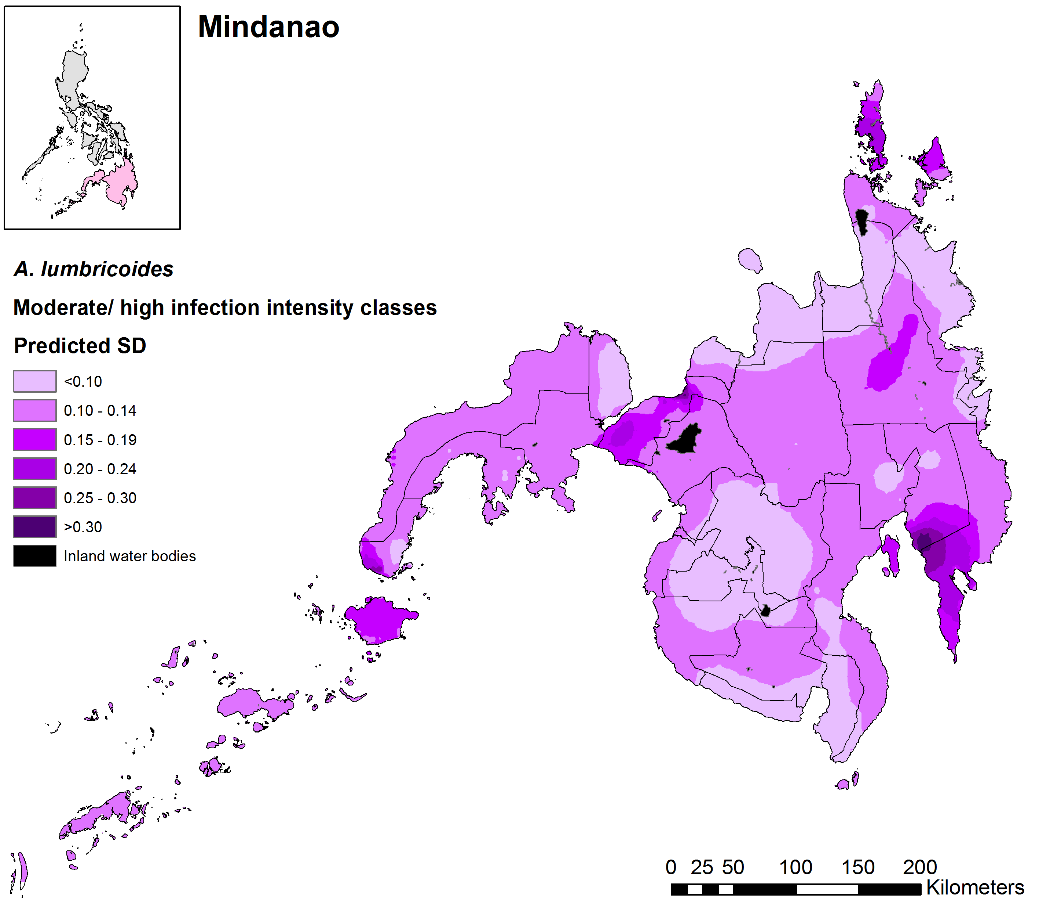


**Figure S9.** Maps of standard deviation (SD) of predicted prevalence of light (**a**) and moderate/high (**b**) infection intensity classes of *A. lumbricoides* in school-aged children in Mindanao, 2017.

**a**


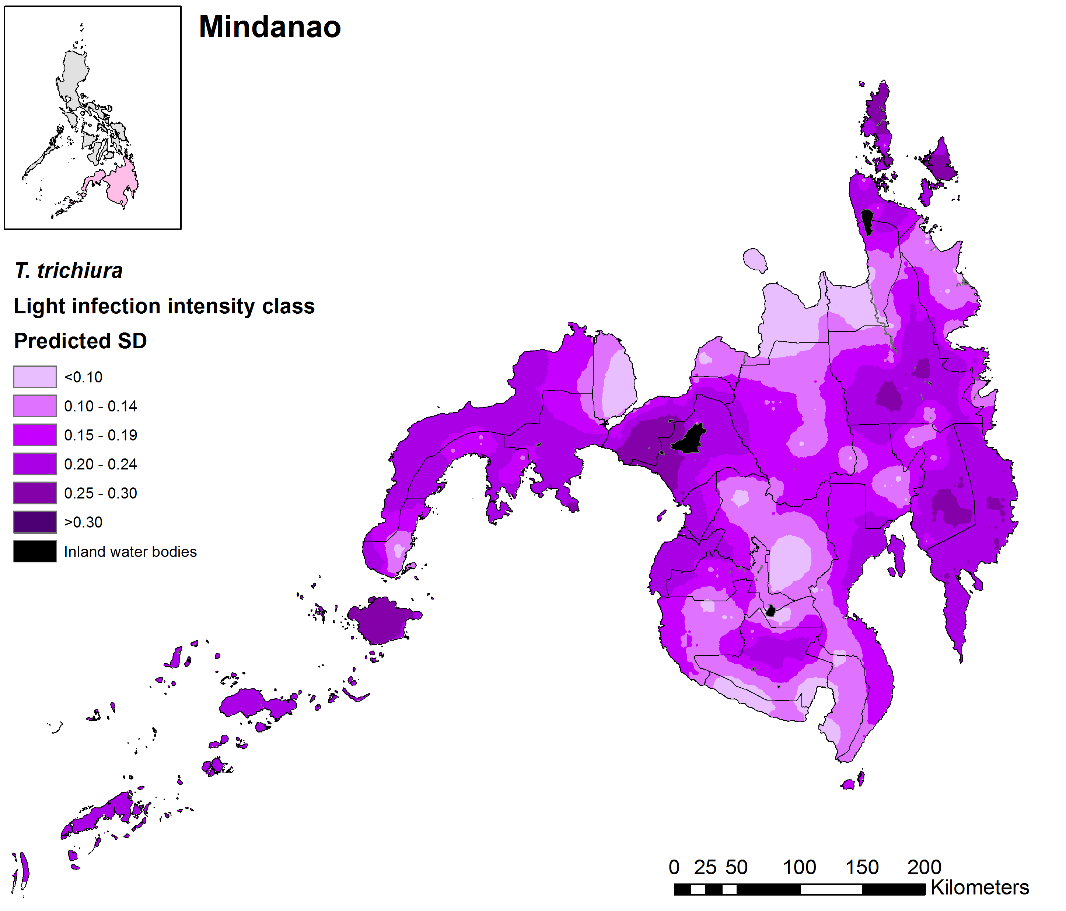


**b**


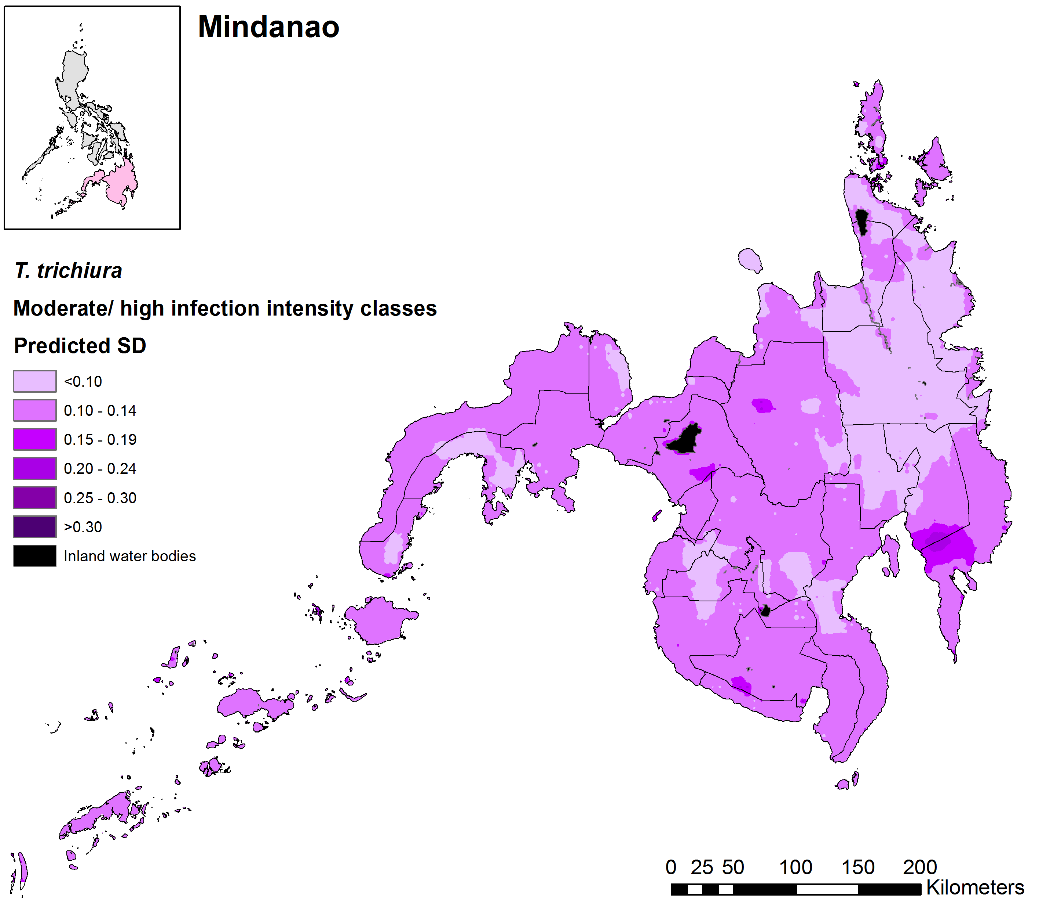


**Figure S10.** Maps of standard deviation (SD) of predicted prevalence of light (**a**) and moderate/high (**b**) infection intensity classes of *T. trichiura* in school-aged children in Mindanao, 2017.


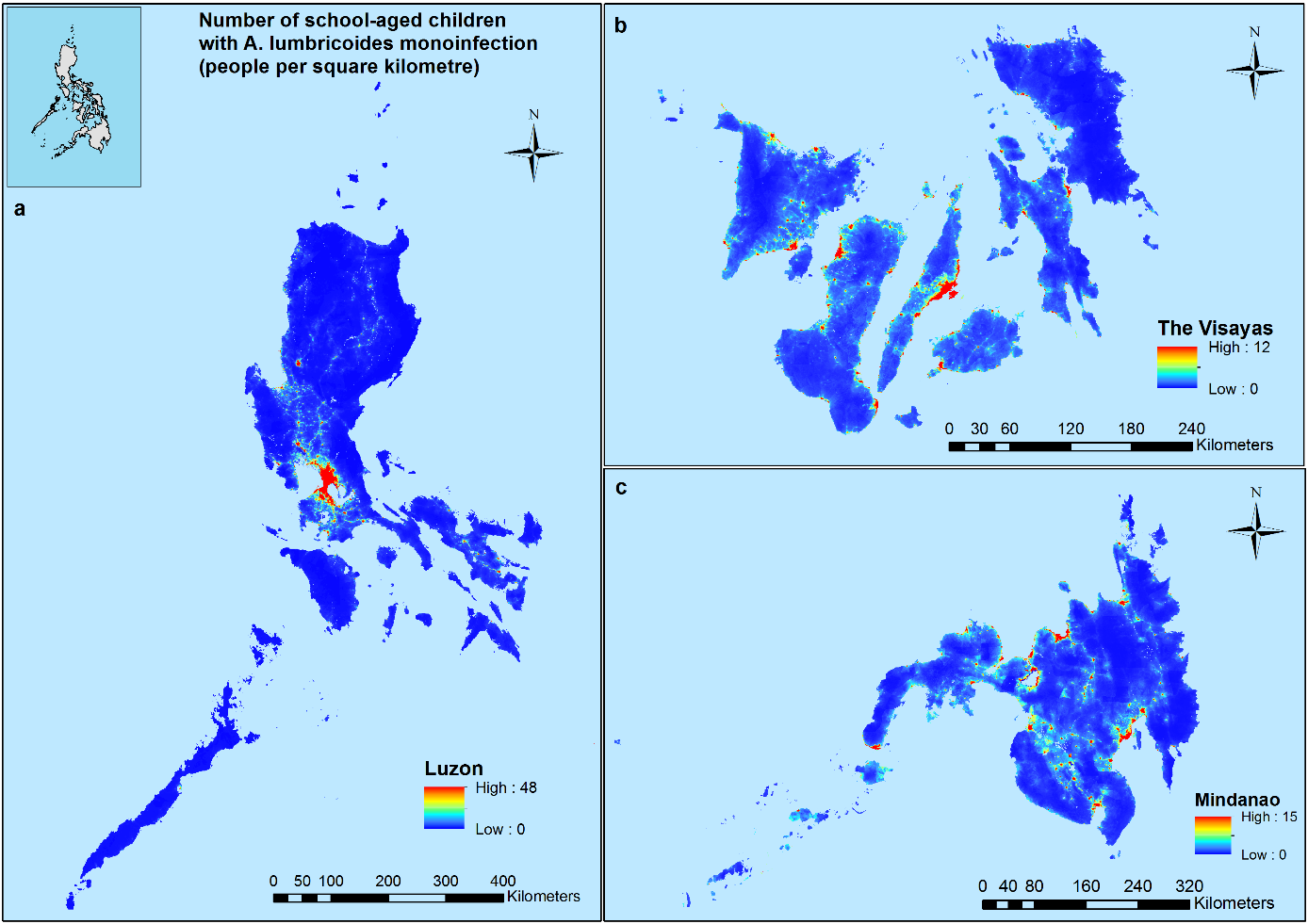


**Figure S11.** Maps showing total number of school-aged children with *A. lumbricoides* monoinfection, people per square kilometre, in the Philippines by region, 2017: Luzon (**a**), the Visayas (**b**) and Mindanao (**c**).


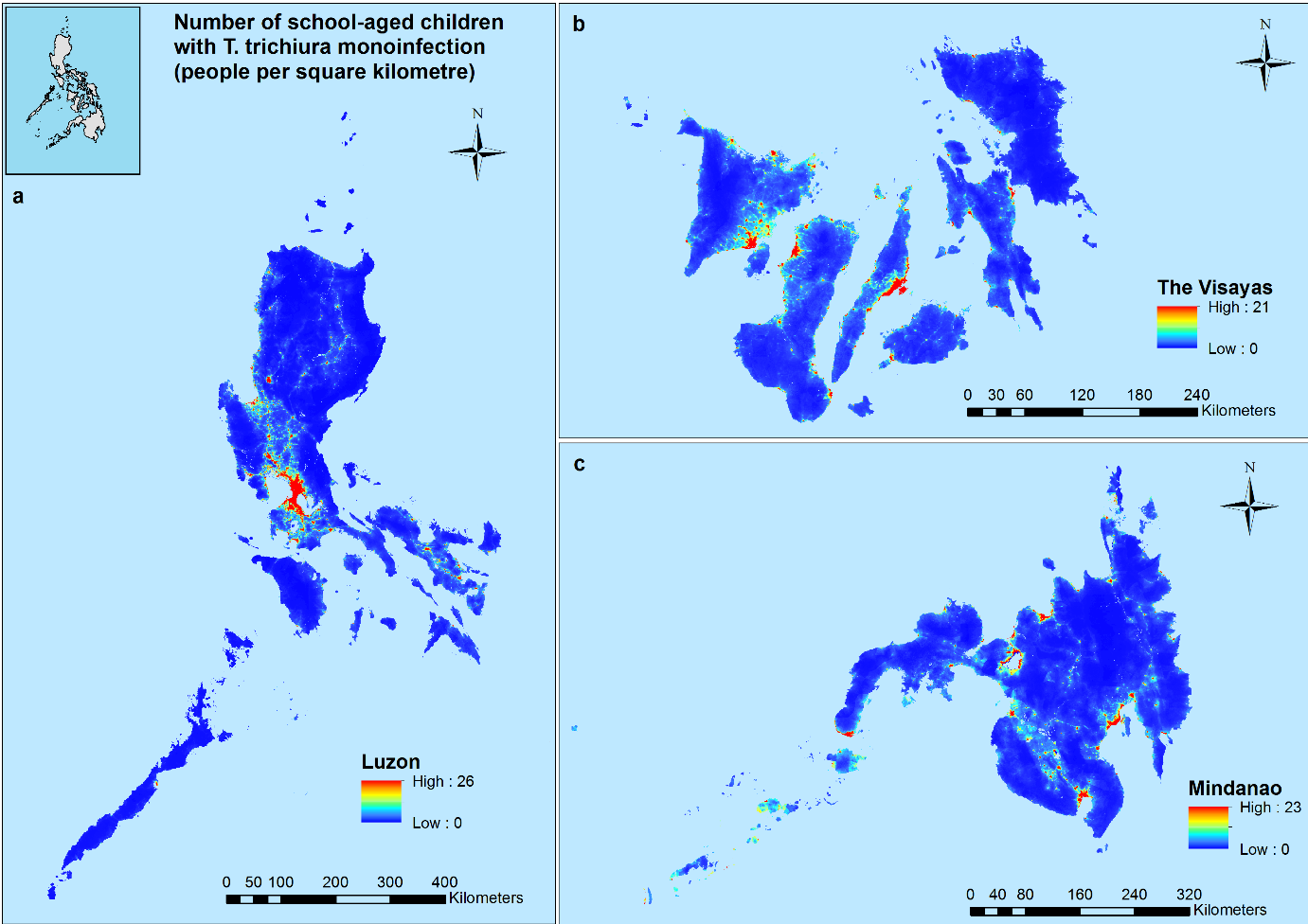


**Figure S12.** Maps showing total number of school-aged children with *T. trichiura* monoinfection, people per square kilometre, in the Philippines by region, 2017: Luzon (**a**), the Visayas (**b**), and Mindanao (**c**).


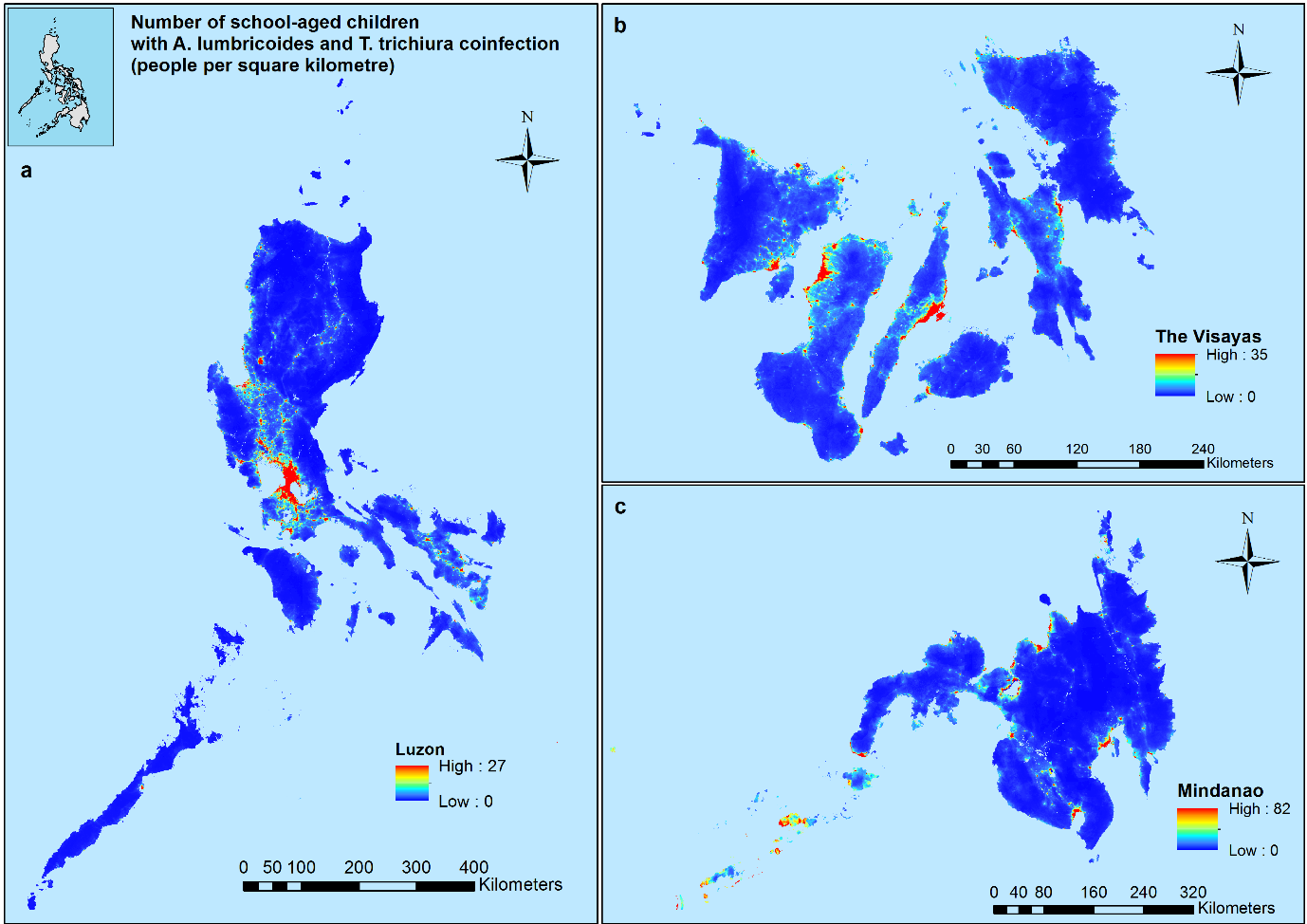


**Figure S13.** Maps showing total number of school-aged children with *A. lumbricoides* and of *T. trichiura* co-infection, people per square kilometre, in the Philippines by region, 2017: Luzon (**a**), the Visayas (**b**), and Mindanao (**c**).

**
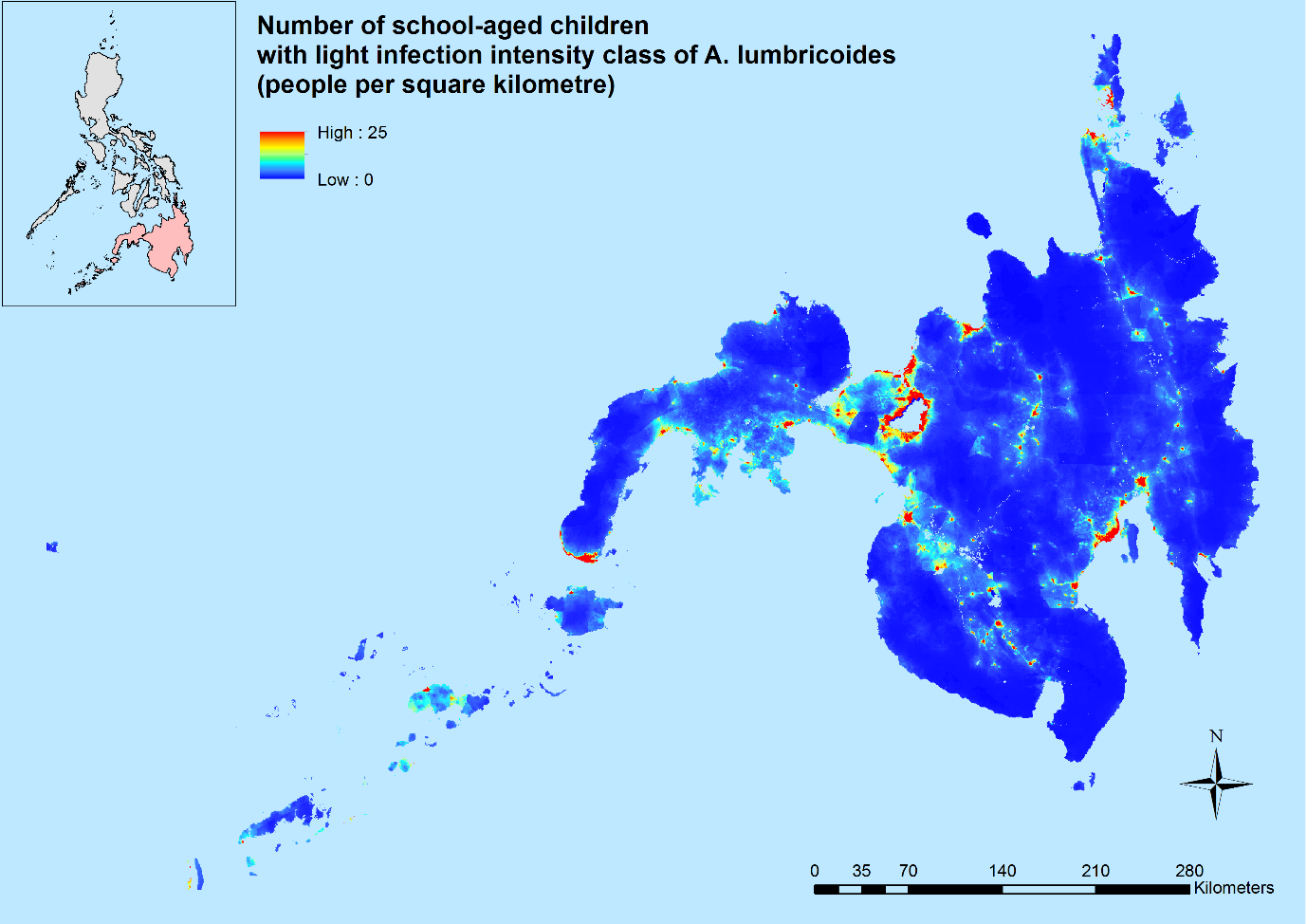
**

**Figure S14.** Map showing a total number of school-aged children infected with light intensity classes of *A. lumbricoides*, people per square kilometre, 2017.

**
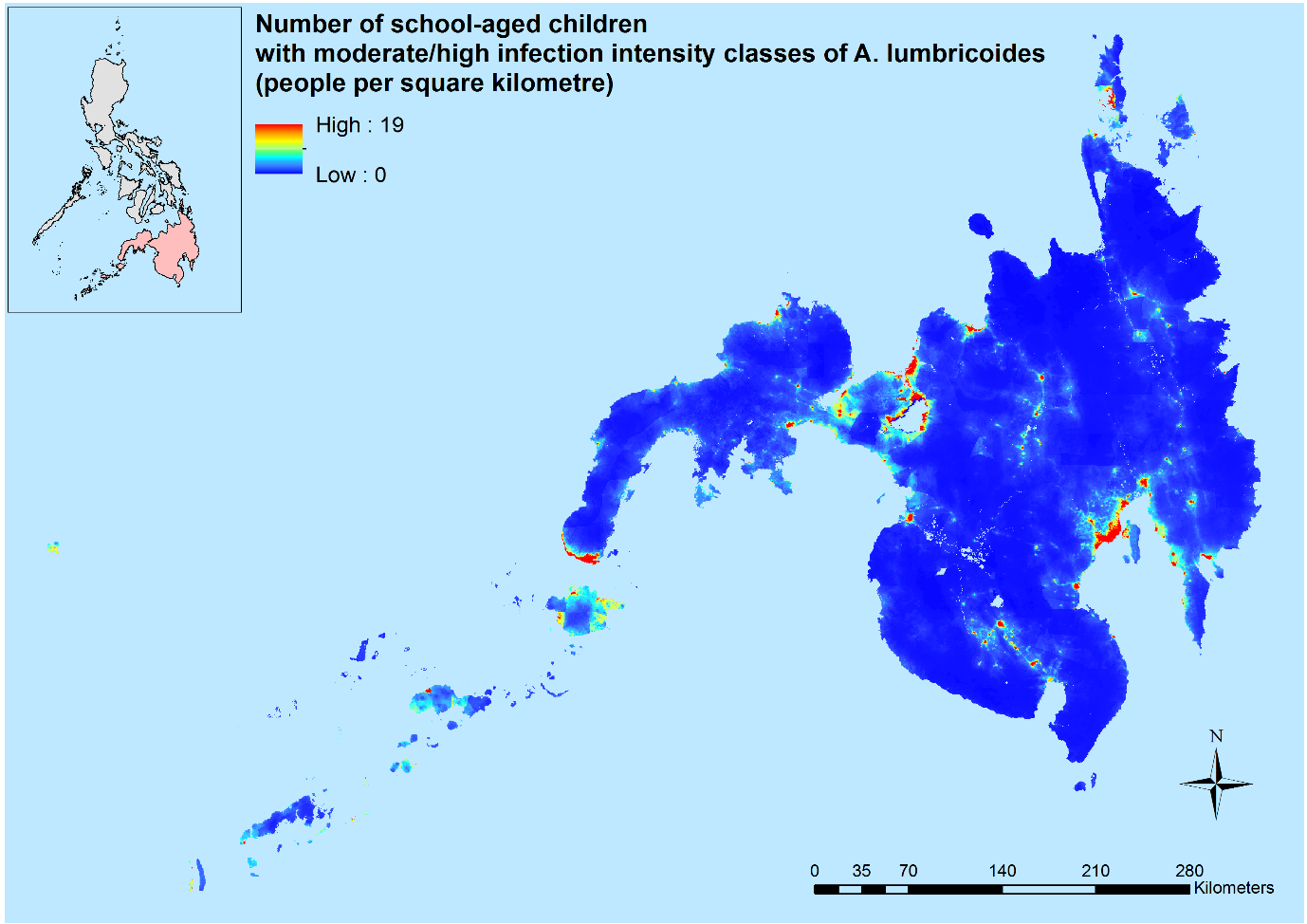
**

**Figure S15.** Map showing a total number of school-aged children infected with moderate/high intensity classes of *A. lumbricoides*, people per square kilometre, 2017.

**
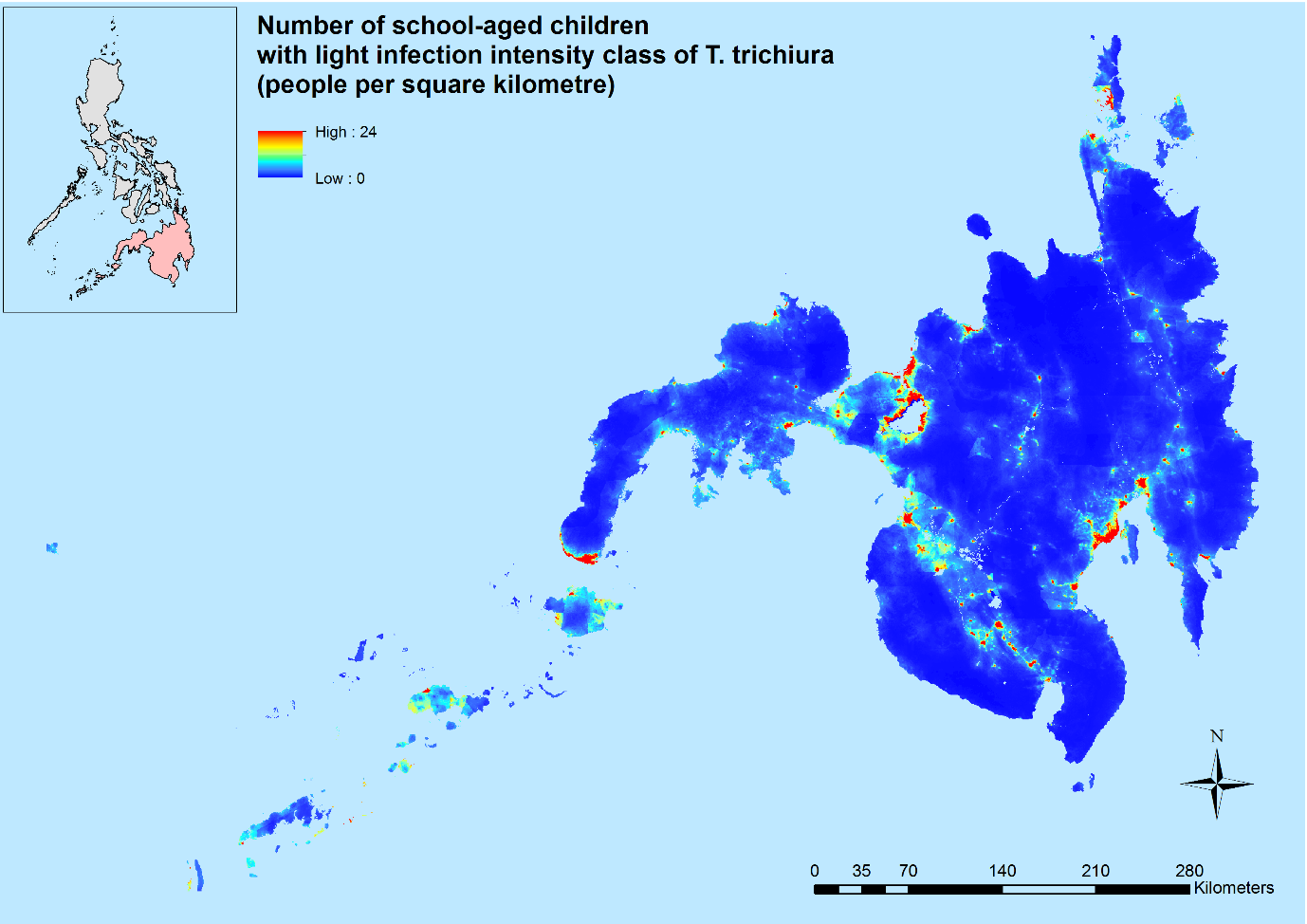
**

**Figure S16.** Map showing a total number of school-aged children infected with light intensity classes of *T. trichiura*, people per square kilometre, 2017.

**
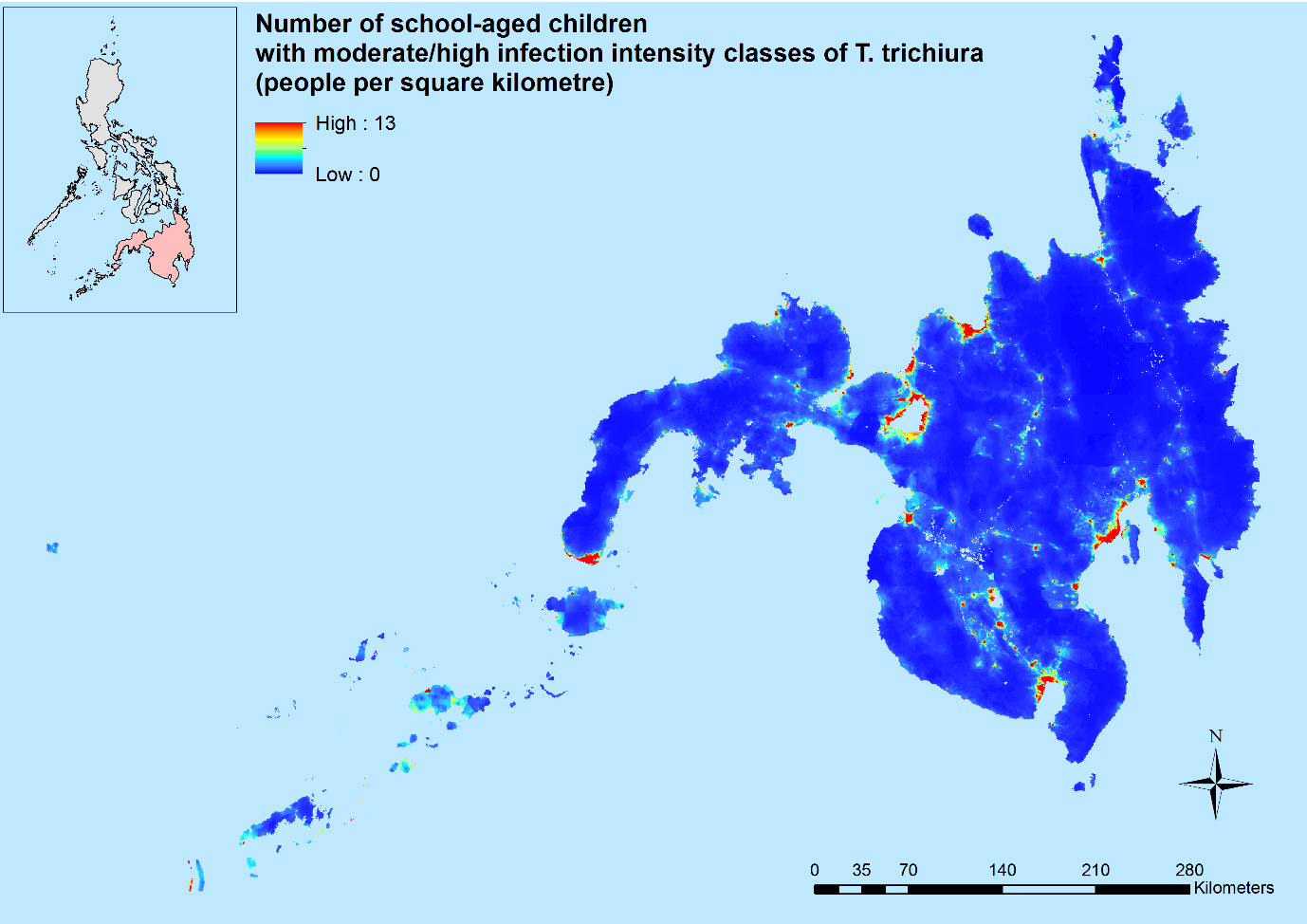
**

**Figure S17.** Map showing a total number of school-aged children infected with moderate/high intensity classes of *T. trichiura*, people per square kilometre, 2017.
